# Powder diffraction and crystal structure prediction identify four new coumarin polymorphs†
†Electronic supplementary information (ESI) available: Crystallographic information files, powder diffraction patterns, lattice constants at 90 K, Hirshfeld surfaces, additional computational details and comparisons. see DOI: 10.1039/c7sc00168a



**DOI:** 10.1039/c7sc00168a

**Published:** 2017-05-15

**Authors:** Alexander G. Shtukenberg, Qiang Zhu, Damien J. Carter, Leslie Vogt, Johannes Hoja, Elia Schneider, Hongxing Song, Boaz Pokroy, Iryna Polishchuk, Alexandre Tkatchenko, Artem R. Oganov, Andrew L. Rohl, Mark E. Tuckerman, Bart Kahr

**Affiliations:** a Department of Chemistry , Molecular Design Institute , New York University , New York City , NY 10003 , USA . Email: shtukenberg@mail.ru; b Department of Physics and Astronomy , High Pressure Science and Engineering Center , University of Nevada Las Vegas , Nevada 89154 , USA . Email: qiang.zhu@unlv.edu; c Department of Geosciences , Stony Brook University , Stony Brook , NY 11794 , USA; d Curtin Institute for Computation and Department of Chemistry , Curtin University , P.O. Box U1987 , Perth , 6845 , Western Australia , Australia; e Department of Chemistry , New York University , New York City , NY 10003 , USA; f Fritz-Haber-Institut der Max-Planck-Gesellschaft , Faradayweg 4–6 , Berlin , 14195 , Germany; g Physics and Materials Science Research Unit , University of Luxembourg , 1511 Luxembourg , Luxembourg; h Department of Materials Science and Engineering , Russell Berrie Nanotechnology Institute , Technion Israel Institute of Technology , Haifa 32000 , Israel; i Skolkovo Institute of Science and Technology , Skolkovo Innovation Center , 3 Nobel St. , Moscow 143026 , Russia; j Courant Institute of Mathematical Sciences , New York University , New York City , NY 10003 , USA; k New York University-East China Normal University Center for Computational Chemistry at NYU Shanghai , 3663 Zhongshan Road North , Shanghai 200062 , China; l Department of Advanced Science and Engineering (TWIns) , Waseda University , Wakamatsucho, 3-2 , Shinjuku , 162-0056 Tokyo , Japan

## Abstract

Coumarin, a simple, commodity chemical isolated from beans in 1820, has, to date, only yielded one solid state structure. Here, we report a rich polymorphism of coumarin grown from the melt. Four new metastable forms were identified and their crystal structures were solved using a combination of computational crystal structure prediction algorithms and X-ray powder diffraction. With five crystal structures, coumarin has become one of the few rigid molecules showing extensive polymorphism at ambient conditions. We demonstrate the crucial role of advanced electronic structure calculations including many-body dispersion effects for accurate ranking of the stability of coumarin polymorphs and the need to account for anharmonic vibrational contributions to their free energy. As such, coumarin is a model system for studying weak intermolecular interactions, crystallization mechanisms, and kinetic effects.

## Abstract 2

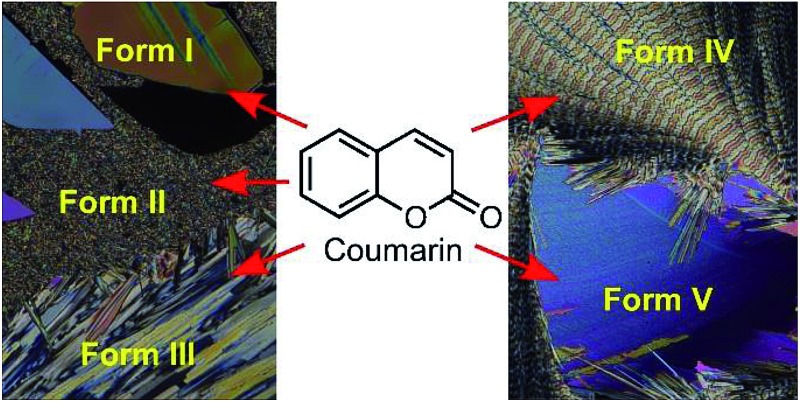
Crystal structures of four new coumarin polymorphs were solved by crystal structure prediction method and their lattice and free energies were calculated by advanced techniques.

## Introduction

Polymorph screening is now recognized as an important step in drug development.^1^ Screenings typically evaluate solution crystallization conditions that yield single crystals amenable to structure analysis by X-ray diffraction. However, crystallization from the melt provides an additional, largely underused opportunity for polymorph screening since it can lead to the creation of large driving forces while suppressing nucleation.^2^ Growth under such conditions frequently leads to polycrystalline mixtures for which structure determination must be coupled with theoretical predictions. The example of coumarin studied here serves to highlight the importance of melt crystallization in polymorph screening, and the necessity in such circumstances of using crystal structure prediction (CSP) in synergy with optical and X-ray crystallography.

**Scheme 1:**
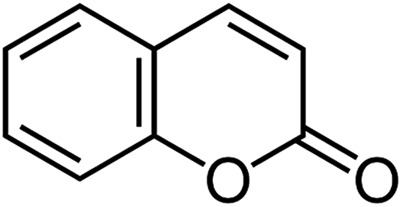
Coumarin, C_9_H_6_O_2_, molecular weight = 146.15 g mol^–1^.

The rich polymorphism of coumarin (Scheme 1), a simple organic compound used in perfumes, medicine, agriculture, and as a precursor for drug synthesis, was broached by Bernauer, who identified two forms in 1929 that crystallized from the melt in the presence of some naturally occurring resins.^3^ Both forms were spherulitic polycrystalline aggregates. Moreover, both forms gave banded spherulites with optical signatures characteristic of ensembles of helically twisted fibrils.^3^ Coumarin initially attracted our attention for this reason.^4,5^ Kofler and Geyr recognized two coumarin forms in 1934 which were identified as monoclinic and orthorhombic on the basis of optical measurements.^6^ Lindpainter recognized three forms with distinct melting points (68.5°, 64.5°, and 55°) in 1939.^7^ However, only one crystal structure is available in the Cambridge Structural Database (CSD).^8–10^

Another form, which turned out to be one of Bernauer's forms, was once again discovered by crystallizing coumarin from the melt in porous poly(cyclohexylethylene) and porous glass bead (diameter of pores 7.5–55 nm) media.^11^ However, its crystal structure was not solved. We repeated Bernauer's crystallization procedures and obtained not one, but four new polymorphs of coumarin. We endeavoured to solve all four new crystal structures and address the following challenges.

(1) Crystal structure determination from a suitable single crystal is nowadays a routine task for X-ray diffraction analysis. However, for many materials only powder X-ray diffraction (PXRD) data are available. Solving crystal structures from PXRD is still a challenge.^12^ Moreover, metastable forms often undergo rapid polymorph conversions and tend to grow concomitantly with other forms, significantly complicating the collection of high quality data required for many crystal structure solution approaches. Alternatively, there has been tremendous progress in the field of crystal structure prediction (CSP) to obtain models of low energy structures.^13,14^ Non-uniqueness of structure solutions solely from PXRD leads to erroneous structures in the literature (*e.g.*, a high pressure phase of Mg(BH_4_)_2_ was initially solved from PXRD,^15^ but later was corrected by CSP^16^). Matching the predicted structures with available, but not necessarily high-resolution, PXRD data provides an alternative way to arrive at the structure. A unit cell delivered by PXRD can delimit CSP, whereas CSP can serve as a check on any structural model developed; iteratively, and in tandem, they work best. Here we use CSP to help solve four new crystal structures of coumarin from PXRD data. Together with the form previously reported in the CSD, the five different forms make coumarin a member of a very small family of multimorphic rigid molecules under ambient conditions.^17^

(2) It is challenging to rank the lattice energies of polymorphs based on theory. The energy differences for organic polymorphs, dominated by intermolecular interactions, are usually within a few kJ mol^–1^. Accuracies within 5 kJ mol^–1^ can now be achieved with van der Waals (vdW) inclusive density functional theory (DFT).^18,19^ Even higher accuracies, within 1 kJ mol^–1^, can be achieved by using computationally demanding wave-function based electronic structure methods but the applicability of these methods to practically relevant molecular crystals is currently limited.^20^ In the absence of strong hydrogen bonds, crystalline coumarin is an ideal system to study vdW interactions. Using data on the newly obtained coumarin polymorphs, we evaluated a variety of vdW-inclusive methods based on DFT and address the importance of many-body interactions. Furthermore, we investigated the finite temperature effect with, and beyond, the harmonic approximation.

## Crystal growth and morphology

Rapid cooling of a coumarin sample melted between two glass slides produces three metastable polymorphs (**II**, **IV**, and **V**). A fourth new polymorph (**III**) was obtained as a product of the transformation of **IV**. These polymorphs are metastable and turn into stable form **I** within a few minutes or even seconds. They, however, can be stabilized by adding 10–30% Canada balsam or some other resins such as Gum mastic. In a mixture with Canada balsam **II**, **III**, and **IV** can survive for a few months and **V** for a few days.

**Fig. 1:**
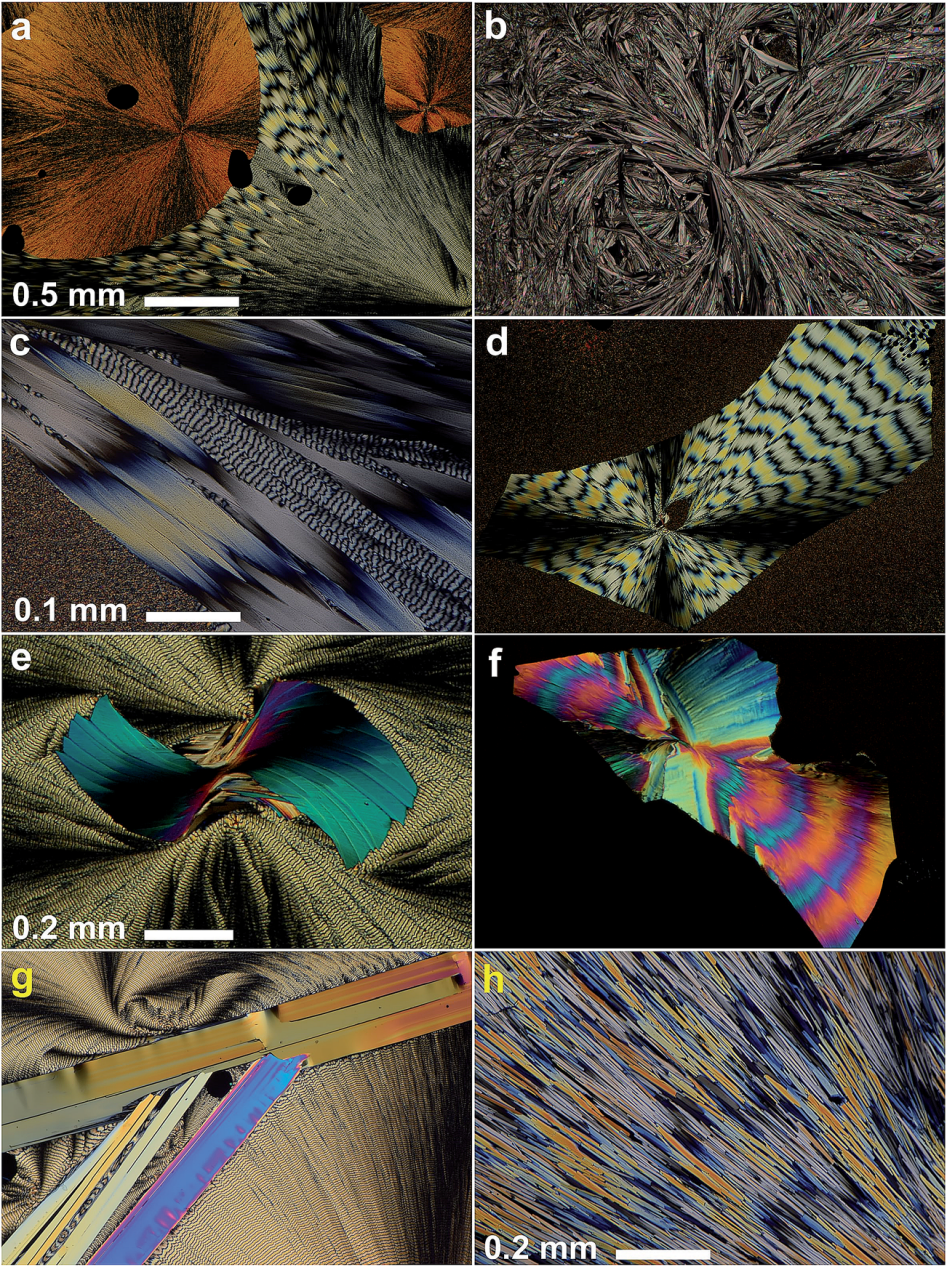
Polarized light optical micrographs of coumarin polymorphs. (a) Spherulites of **II** showing a Maltese cross embedded into banded spherulites of **IV** formed at ∼22 °C. (b) Spherulite of **II** formed at ∼40 °C. (c) Banded spherulite of **IV** showing two twist periods. Left lower corner – form **II**. Growth at ∼22 °C. (d) Banded spherulite of **IV** formed at ∼22 °C and surrounded by **II**. (e) Crystals of **V** surrounded by **IV**, the latter was formed in the course of cross-nucleation. Growth at ∼22 °C. (f) Banded spherulite of **V** surrounded by **II**. Growth at ∼40 °C. (g) Large crystals of **III** formed from **IV** at 54–57 °C and surrounded by banded spherulites of **IV** that later crystallized at room temperature. (h) Banded spherulites of **IV** fully replaced by needle-like crystals of **III** at 56 °C. Note that the banding is still visible. In figures (b), (d), (f), (g) the scale bar is the same as in (a). All samples were obtained from coumarin mixtures with Canada balsam (21 wt% for (c), (g), and (h); 20–40 wt% for the rest).

Coumarin **II** spontaneously crystallizes at 4–50 °C. At higher temperatures, nucleation does not occur, whereas 4 °C is the lowest temperature at which experimental data was obtained. **II** forms spherulites consisting of irregular, curved, and highly birefringent crystallites, whose size increases with temperature (Fig. 1a and b). Below 31 and 35 °C, **II** is accompanied by spontaneously nucleated **IV** and **V**, respectively.

**Fig. 2:**
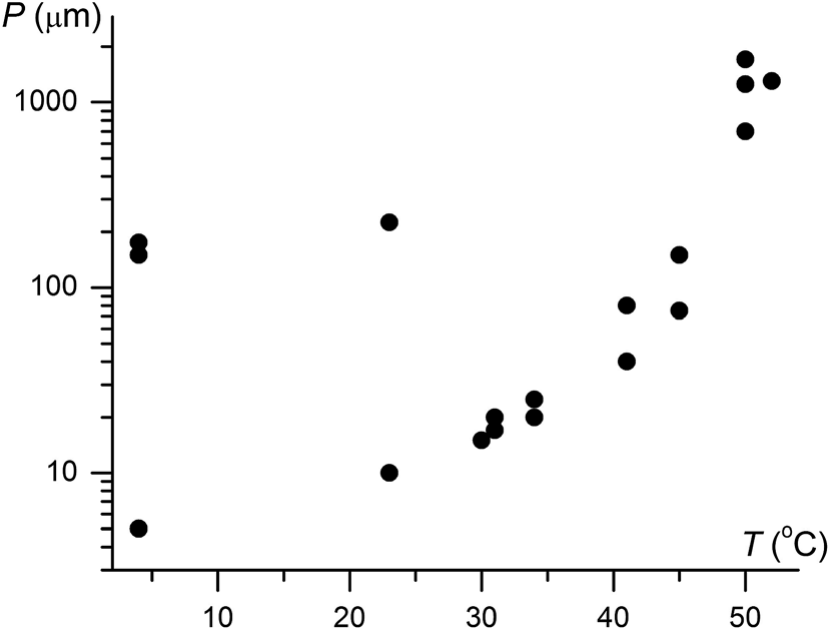
Twist period (pitch, *P*) of coumarin **IV** crystallized in the presence of 21 wt% Canada balsam as a function of growth temperature, *T*. Note two very different coexisting pitches below 25 °C.

Although **IV** does not nucleate above 31 °C, it can crystallize at higher temperatures by seeding. In the whole temperature range, it forms banded spherulites (Fig. 1a, c and d) with the twist period or pitch (π rotation of the fiber around the growth direction) increasing with temperature (Fig. 2). Such behaviour is typical for the banded spherulites and should be related to thinner crystallites and higher driving forces for crystallization at lower temperatures.^4^ Spherulites alternate between optically positive (slow direction radial) and negative (slow direction tangential) along the radii, indicating that the intermediate refractive index *N*_*Y*_ is radial and that the minimum (*N*_*X*_) and maximum (*N*_*Z*_) refractive indices are exchanged as the radii twist, thereby forming concentric bands of optical contrast between crossed polarizers (Fig. 1a, c and d). At temperatures below 25 °C, two twist periods can coexist within one spherulite (Fig. 1c and 2), a rare and puzzling behaviour that was reported before for banded spherulites of *ε* resorcinol.^14^

Coumarin **V** is a minor form, whose largest fraction (up to 10%) was obtained at ∼30 °C; at higher temperatures the nucleation rate is too low whereas at lower temperatures **V** is easily replaced by **IV** *via* cross-nucleation events (Fig. 1e). Coumarin **V** forms relatively large crystallites that sometimes organize themselves into spherulites. The spherulites can also be banded with twist periods greater than 0.3 mm (Fig. 1f) and maximum refractive index *N*_*Z*_ oriented radially.

Among all five forms, twisted crystals have been observed for coumarin **IV** and **V** only. Form **I** does not crystallize as fine needles are typically required for twisted morphologies. The reasons for the presence of twisted morphologies in **IV** and **V** and its absence in **II** and **III** are not clear. As demonstrated by the aggregate of experimental data, twisting does not seem to be directly related to the crystal structure, so that different polymorphs of the same material can show twisted and non-twisted morphologies.^4^ However, if **IV** or **V** are twisted, the other crystals of the pair are likely twisted too.

**Fig. 3:**
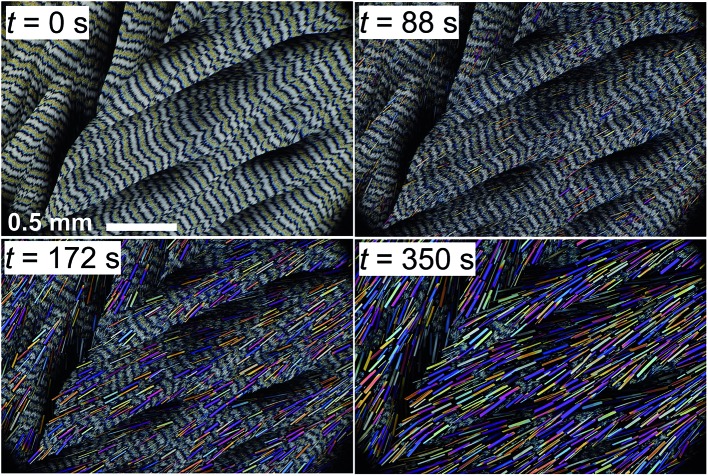
Phase transformation of coumarin **IV** to **III** at 57 °C observed with a polarized light optical microscope. Concentration of Canada balsam 21 wt%.

Form **IV** held at *T* > 50 °C for a few minutes transforms into **III**; prismatic crystals (Fig. 1g) form that are elongated parallel to the elongation of fibers in the original spherulite (Fig. 3). Comparison of interference colors also suggests some correspondence between crystallite orientations of these phases in the perpendicular plane (Fig. 1h).

**Fig. 4:**
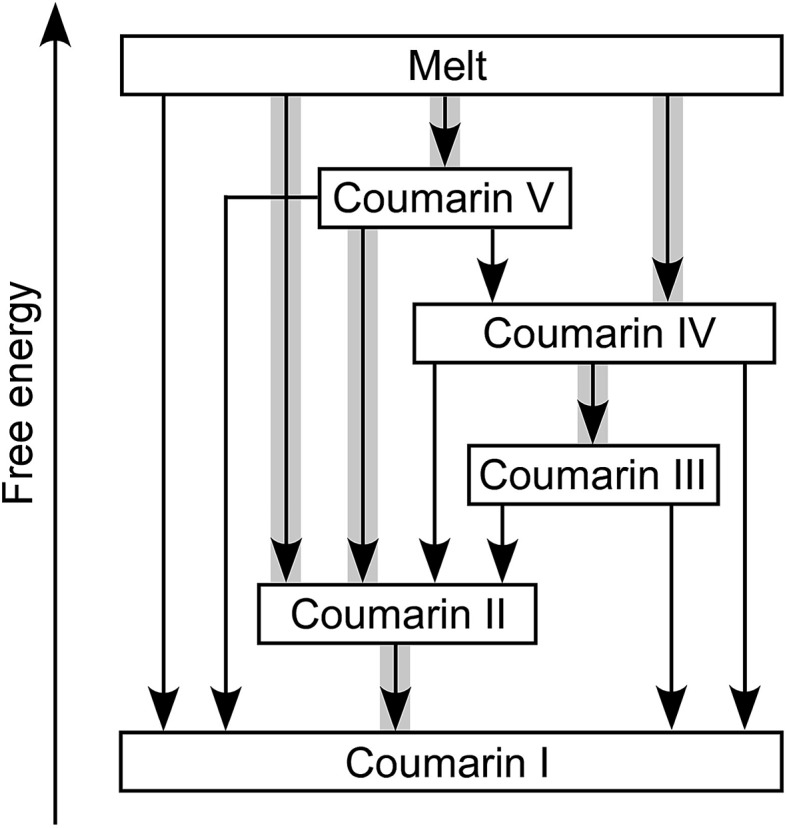
Relationships among coumarin polymorphs at and above room temperature. Arrows correspond to transformation *via* motion of an interface. Thicker gray lines highlight transformations where nucleation of a new phase was also detected.

Coumarin **II** transforms to **I** by nucleation and growth. Transformation of other forms usually occurs *via* motion of the growth front of **I** nucleated elsewhere, often in the course of coumarin sublimation and recrystallization. Form **II** can directly nucleate and grow inside **V**. Coumarin **III** can also grow inside **V** if they are in close contact. Likewise, **II** can grow into **III** and **IV**. These phase relationships were observed at and above room temperature (Fig. 4). The free energy ranking obtained from these relationships (**I** < **II** < **III** < **IV** < **V**) is corroborated with the ranking obtained from melting temperatures (Table 1).

**Table 1:** Melting points of coumarin polymorphs^*a*^

Polymorph	Melting point, *T*_m_, °C			Δ*G* ^*b*^, kJ mol^–1^
	No additive	No additive, ref. 11	Canada balsam, 21 wt%	
Coumarin **I**	69.7(2)	71	63.9(10)^*c*^	0
Coumarin **II**	66.2(2)	n/d	59.4(5)	0.19
Coumarin **III**	66.0(2)	n/d	61.3(2)	0.20
Coumarin **IV**	64.9(7)	65	59.4(10)^*c*^	0.26
Coumarin **V**	n/d	n/d	50.9(20)^*c*^	∼0.84^*d*^

^*a*^n/d – not determined.

^*b*^Difference in free energy at *T*_m(_ **_I_**_)_ Δ*G* = (*T*_m(_ **_I_**_)_ – *T*_m_)Δ*H*/*T*_m(_ **_I_**_)_, where the heat of fusion Δ*H* = 18.4 kJ mol^–1^.

^*c*^An accurate value of *T*_m_ is hard to establish because of coumarin dissolution in Canada balsam.

^*d*^ *T*_m_ = 54 °C was estimated by comparing differences *T*_m(_ **_I_**_)_ – *T*_m_ measured with and without Canada balsam. Based on the melting points, polymorphs **II**, **IV**, and **V** were presumably discovered by Lindpainter.^7^

**Fig. 5:**
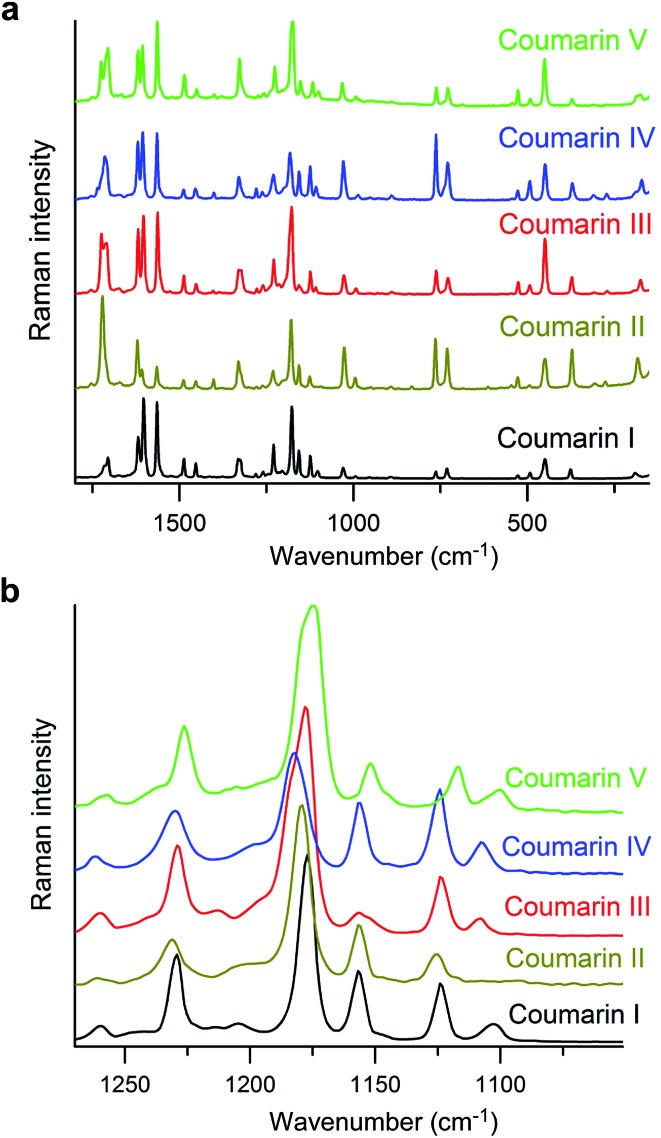
Raman spectra of coumarin polymorphs. (b) Enlarged segment of (a) emphasizing spectral differences.

Measurement of melting points, *T*_m_, using differential scanning calorimetry (DSC) was impossible for any form except **I** due to fast polymorph transformation. Consequently, the melting points were measured with a hot stage (Table 1), and the data obtained from DSC (*T*_m_ = 69.7 °C; heat of fusion, Δ*H* = 18.4 kJ mol^–1^, (lit. 17.2(4) kJ mol^–1^ (ref. 11))) were used for calibration of the *T*_m_ obtained with a hot stage. Although the values of *T*_m_ for coumarin crystallized in the presence of Canada balsam are shifted with respect to coumarin without additives, the differences between melting points of different polymorphs are comparable and can serve as a measure of the free energy difference between polymorphs. The micro-Raman spectra of coumarin polymorphs are similar (Fig. 5a) and close to the spectra reported for coumarin solutions.^21^ Nevertheless, all polymorphs can be distinguished by the distinct signatures in the Raman spectra (Fig. 5b).

## Crystal structure solution

All the new polymorphs crystallized from the melt in polycrystalline form. Preliminary data collection for all polymorphs was carried out at room temperature in reflection mode using a microdiffractometer equipped with a 2D detector. Room temperature and 90 K high-resolution powder data were recorded for **II**, **III**, and **IV** on the ID22 beamline at the European Synchrotron Research Facility (ESRF).

With only the structural data for the polycrystalline samples from PXRD, polymorphism of coumarin was explored using two independent CSP methods. To solve the unknown crystal structures, we performed a systematic crystal structure search with evolutionary algorithms for structure generation and DFT energy ranking (called CSP_A_ in this work). This search was complemented by a second CSP method employing a classical force field (called CSP_B_).

To solve the unknown crystal structures within the CSP_A_ protocol, we performed a systematic crystal structure search based on the evolutionary algorithms implemented in the USPEX code.^22–25^ The most significant feature of this approach is that molecular geometry is the only structural input. The number of asymmetric units (*Z*′) and choices of space groups, specified by the user, define the extent of the crystal structure search. Optionally, one can set the unit cell, if the lattice constants are known. The DMACRYS code^26^ was use to perform the structure relaxations within USPEX. In DMACRYS, the distributed multipole analysis model was constructed by using the calculated Møller–Plesset MP2/6-31G(d,p) charge density from Gaussian09 (ref. 27) and the FIT^28^ empirical repulsion-dispersion potentials.

**Fig. 6:**
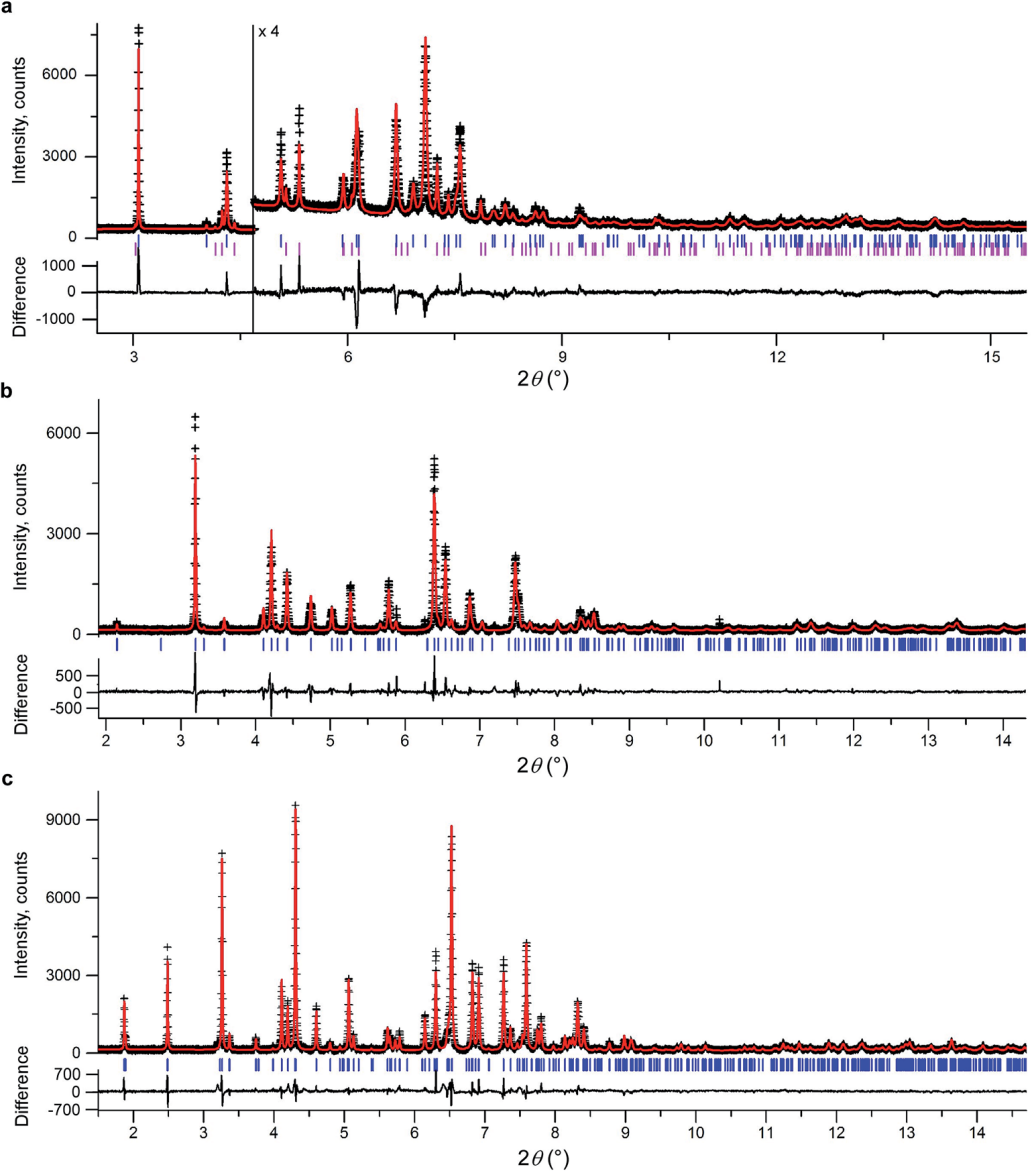
Rietveld refinement of high-resolution synchrotron powder diffraction data for a powder sample of **II** (a), **III** (b), and **IV** (c). All samples contain 21 wt% Canada balsam. The data were collected at the ESRF at a wavelength of 0.41064(1) Å (a) and 0.39992(1) Å (b and c) and at room temperature (a) and 90 K (b and c). Observed intensities – black crosses, calculated intensities – red lines. Blue ticks are reflection positions. Magenta ticks in (a) mark reflection positions for **I** (the calculated fraction of **I** is 17.8 wt%). The lower traces show the difference curves.

**Fig. 7:**
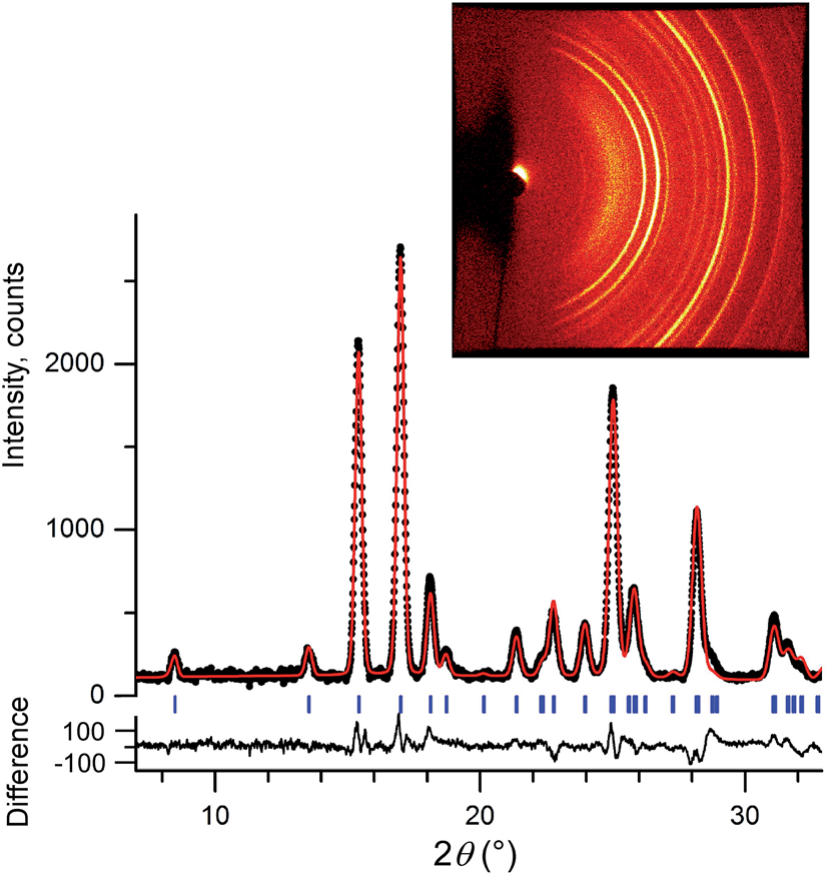
Room temperature 2D diffraction pattern (inset) and corresponding integrated intensities of a powder sample of **V** (black dots) along with the calculated pattern (red line). Sample contains 21 wt% Canada balsam. The data were collected with a Bruker D8 DISCOVER GADDS microdiffractometer at room temperature using Cu-Kα radiation. Blue ticks are reflection positions. The lower trace shows the difference curve.

We initially conducted a blind search for coumarin crystal structures with *Z*′ = 1 and 2 for the 30 most common space groups, similar to blind test conditions.^13^ Among the 100 low-energy structures, we immediately found two models that matched the experimental PXRD of **II** (Fig. 6a) and **V** (Fig. 7). PXRD calculated for predicted structures were visually compared with experimental PXRD data, and the lattice constants and peak profiles were refined using a Rietveld method for the promising candidates to figure out if a match occurred.

However, no matches for **III** and **IV** were obtained. Therefore, we determined the lattice constants using the indexing software McMaille v3.04.^29^ The calculated unit cell for **IV** was found to be orthorhombic with *a* = 14.220(5), *b* = 6.025(2), *c* = 24.792(6) Å. For **III**, single crystals with typical sizes 2 mm × 0.15 mm × 5 μm (Fig. 1g) were obtained by recrystallizing **IV** between two glass slides at 54–58 °C for an hour. With the help of a microdiffractometer equipped with a 2D detector, we collected about 40 reflections, determined their corresponding diffraction vectors,^30^ and found an orthorhombic unit cell with *a* = 13.79(10), *b* = 6.02(7), *c* = 16.98(12) Å. Using these parameters, we performed two independent structure searches with these cells, namely *Z*′ = 2 for **III** and *Z*′ = 3 for **IV**, for the common space groups *P*2_1_/*c*, *P*2_1_2_1_2, *P*2_1_2_1_2_1_, *Pca*2_1_, and *Pna*2_1_. The lowest-energy structures from the fixed cell searches matched the experimental PXRD patterns (see Fig. 6b and ESI Fig. S1 for **III** and Fig. 6c and ESI, Fig. S2† for **IV**, respectively). We then repeated the prediction for the same space groups without specifying cell parameters for both *Z*′ = 2 and *Z*′ = 3. Forms **III** and **IV** were identified in each search, confirming that the results obtained from fixed-cell optimizations are indeed low-energy structures. Interestingly, we also found that the comparison of diffraction patterns confirms that the metastable form reported in ref. 11 corresponds to **IV**.

After finding the candidate structure models, the lattice constants were refined using the Rietveld method implemented in the FullProf suite^31^ (Table 2 and ESI, Table S1†). For **II** (room temperature), **III** (*T* = 90 K and room temperature), **IV** (*T* = 90 K and room temperature), and **V** (room temperature) the atomic coordinates were also refined by fixing coumarin molecules as rigid bodies using the FullProf suite and Bruker TOPAS 5 (ref. 32) software; final cif files are listed in ESI† and agreement factors are shown in Table 3. In order to check whether the refinement leads to significant structural change, the models before and after refinement were expanded to clusters consisting of 20 molecules and then compared using the COMPACK algorithm.^33^ The calculated root mean-squared deviation (RMSD) values are generally very small, <0.3 Å (Table 3), confirming the excellent agreement between experiment and theory.

**Table 2:** Comparison of the structures of coumarin polymorphs (room temperature data; data collected at 90 K are summarized in ESI, Table S1)

Polymorph	Coumarin **I** ^9^	Coumarin **II** ^*a*^	Coumarin **III** ^*a*^	Coumarin **IV** ^*a*^	Coumarin **V** ^*b*^
Space group	*Pca*2_1_	*P*2_1_	*P*2_1_2_1_2_1_	*P*2_1_2_1_2_1_	*P*2_1_2_1_2_1_
*a* (Å)	15.5023(11)	3.980	17.066	24.722	4.868
*b* (Å)	5.6630(4)	15.291	6.038	5.994	6.882
*c* (Å)	7.9102(6)	5.858	13.888	14.310	20.851
*β* (°)	90	85.76	90	90	90
*V* (Å^3^)	694.4	355.5	1431.0	2120.5	698.4
*Z*, *Z*′	4, 1	2, 1	8, 2	12, 3	4, 1

^*a*^Data collected at ESRF.

^*b*^Data collected with a microdiffractometer. Reported errors from least squares fitting of lattice parameters (1–2 × 10^–4^ Å) are too small to be physically meaningful.

**Table 3:** Agreement factors and RMSD for all the structures analyzed

Polymorph	*T*, K	Experiment^*a*^	CCDC code/dep. number	*N* ^*b*^	*R*_p_, %	*R*_wp_, %	*R*_exp_, %	*χ* ^2^	RMSD, Å^*c*^
Coumarin **I**	90	Ref. 9	COUMAR11	n.r.	2.43	2.43	n.r.	n.r.	0.123
	295	Ref. 10	COUMAR12	n.r.	3.62	3.62	n.r.	n.r.	0.173
Coumarin **II**	298	ESRF	1542946	130	6.97	9.67	6.49	2.22	0.198
Coumarin **III**	90	ESRF	1542947	849	9.82	12.79	9.44	1.83	0.234
	298	GADDS	1542948	49	4.74	6.49	2.87	5.11	0.264
Coumarin **IV**	90	ESRF	1542949	1013	10.41	12.63	9.09	1.93	0.198
	298	ESRF	1542950	1008	13.35	15.68	7.64	4.22	0.244
Coumarin **V**	298	GADDS	1542951	79	8.09	10.82	6.07	3.18	0.295

^*a*^ESRF – high-resolution PXRD data obtained at the synchrotron; GADDS – low-resolution PXRD data collected with a laboratory diffractometer.

^*b*^ *N* – number of reflections.

^*c*^RMSD – root mean-squared deviation of CSP structure and the structure refined on experimental data. n.r. – not reported.

**Fig. 8:**
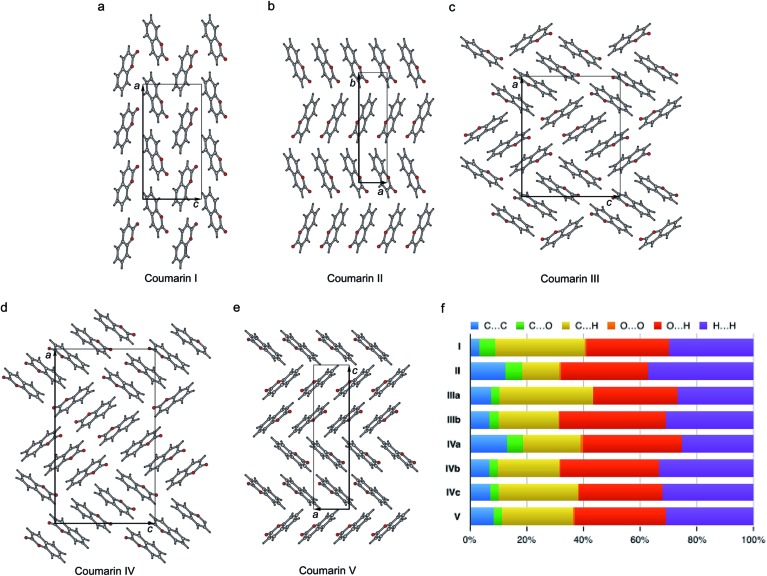
Crystal structures of coumarin polymorphs **I** (a), **II** (b), **III** (c), **IV** (d), and **V** (e) and the percentage contributions to Hirshfeld area for the close intermolecular contacts (f).

The crystal structures of all five polymorphs are shown in Fig. 8 and summarized in Table 2 and ESI, Table S1.† Four of the five polymorphs are orthorhombic, while **II** is monoclinic. The most stable **I** (space group *Pca*2_1_) adopts a herringbone motif in the *bc*-plane. In the four other structures, coumarin molecules form stacks with molecular planes separated by 3.3–3.6 Å. In **II** and **V**, there are infinite stacks running parallel to the *c* and *a* directions, respectively. The major difference between these two structures is how the stacks alternate along the *b* and *c* axes. In **II**, there are two types of stacks with different orientations of molecules with respect to a common coordinate system. In **V** there are four such types forming two pairs with similar molecular orientations.

Coumarin **III** and **IV** belong to the orthorhombic space group *P*2_1_2_1_2_1_ but differ in the number of asymmetric units, *Z*′ = 2 and 3, respectively. They are characterized by similar parquet-like arrangement of stacks in the *ac*-plane, each containing four and six molecules, respectively. Similarity of molecular packing and closeness in lattice constants *b* and *c* can explain orientational relationships between **III** nucleating over **IV** with coinciding *b* axes and some correspondence in the *ac*-plane (Fig. 1h and 3).

In parallel to the CSP_A_ scheme described above, a second CSP approach (CSP_B_) was employed to compare structure generation techniques, test the reliability of an OPLS-based classical force field, and check the thermal stability of predicted structures. In this approach, random structures were generated *via* the UPACK program suite^34^ using a rigid molecule with geometry from a PBE0/6-311G*^35,36^ DFT optimization in Gaussian09.^27^ A modified OPLS force field^37^ in which ESP charges were determined based on the computed electron density was used for energy evaluations. In the initial stage of CSP_B_, 1000 structures were generated for *Z*′ = 1 and 2 in each of the 13 space groups most common for organic molecules (*P*2_1_/*c*, *P*1, *P*2_1_2_1_2_1_, *P*2_1_, *Pbca*, *C*2/*c*, *Pna*2_1_, *Cc*, *Pca*2_1_, *C*2, *P*1, *Pbcn*, *Pc*), with an external pressure of 1 bar. This search generated 58 unique structures within 5 kJ mol^–1^ of the lowest energy, which corresponds to coumarin **I**. The initial set of predicted structures also included **II**, **III**, and **V** within 7 kJ mol^–1^ of form **I**. A subsequent search with 5000 structures for each of the 13 space groups and using *Z*′ = 1, 2, and 3 resulted in a total of 104 unique structures found within 5 kJ mol^–1^ of **I**. Using a dedicated search with *Z*′ = 3 in the *P*2_1_2_1_2_1_ space group, coumarin **IV** was generated only once in 60 000 random structures. However, many structures, including the observed forms with *Z*′ = 1 or 2, were predicted by both CSP methods (see ESI†) and are discussed further below.

To test the thermal stability of the generated structures, the observed polymorphs and 20 other low-energy structures were equilibrated using molecular dynamics (MD). For this subset of possible polymorphs, these simulations were performed at 300 K and 1 bar *via* flexible-cell isothermal–isobaric MD (see Methods for details). After an expected thermal expansion of the cell volumes (<5% for observed polymorphs), all structures tested were found to be stable under these conditions.

As reported in Table 1 and Fig. 4, experimental observations indicate that the order of stability for the five coumarin polymorphs is **I** > **II** > **III** > **IV** > **V**, which was not observed in the final energy ranking for either CSP method. It is well known that the energy ranking of predicted crystal structures remains challenging.^13^ To explore the performance of different ranking methods on a set of polymorphs, an extensive analysis based on DFT and free energy calculations is reported below.

## Lattice energy landscape and Hirshfeld surface

Crystal polymorphism originates from the competition between intramolecular and intermolecular interactions for different crystal packings. There have been tremendous efforts in developing accurate methods to describe vdW interactions in the framework of DFT in recent years.^18,19,38–40^ To assess the performance of different DFT models in molecular crystals, the so-called C21 test set was proposed^41^ and subsequently extended to the X23 reference set.^42^ Since high-level benchmark calculations are not available for a variety of molecular crystals, the reference geometries of experimentally determined crystal structures and experimental sublimation enthalpies, which have been back-corrected for vibrational contributions, serve as benchmarks. Since various methods have been shown to achieve good accuracy in lattice energies (within 5 kJ mol^–1^ of mean absolute errors) and unit cell geometries,^18^ the newly obtained set of coumarin polymorphs provides an ideal test for evaluating the performance of electronic structure methods.

In order to account for the missing long-range interactions in standard DFT, various methods have been proposed to explicitly incorporate vdW interactions. One common approach is to add, a *posteriori*, an energy term of the general form of –*C*_6_/*R* ^6^, which describes a pairwise level the first term of vdW interactions between two dipoles in a multipole expansion. The *C*_6_ term represents the dipole–dipole dispersion coefficient between the two atoms involved and *R* is the interatomic distance. This scheme is used for example in Grimme's DFT-D^43^ and DFT-D2 (ref. 44) methods (using fixed empirical dispersion coefficients), and by the Tkatchenko–Scheffler (TS)^45^ method, in which the dispersion coefficients are explicitly dependent on the electron density. The DFT-D3 scheme^46^ includes in addition, dipole-quadrupole terms and optionally also three-body dipolar interactions, while the exchange-dipole moment (XDM) methods^41^ treat vdW interactions on a pairwise level up to quadrupole–quadrupole contributions. Another approach is to obtain dispersion interactions by designing functionals that explicitly include nonlocal correlations (though still based on pairwise addition), such as vdW-DF,^47^ vdW-DF2 (ref. 48), and their empirically optimized versions (optB88 and optPBE).^19^ Furthermore, Tkatchenko and coworkers proposed the many-body dispersion (MBD) method,^49^ which describes many-body dipolar interactions up to infinite order and also includes electrodynamic response effects. It was found that the MBD method substantially outperforms the original TS scheme, in particular for molecular crystals.^50^ In addition, it was found that the use of MBD together with a hybrid functional can be necessary for obtaining correct stability rankings for molecular crystals.^51^

**Fig. 9:**
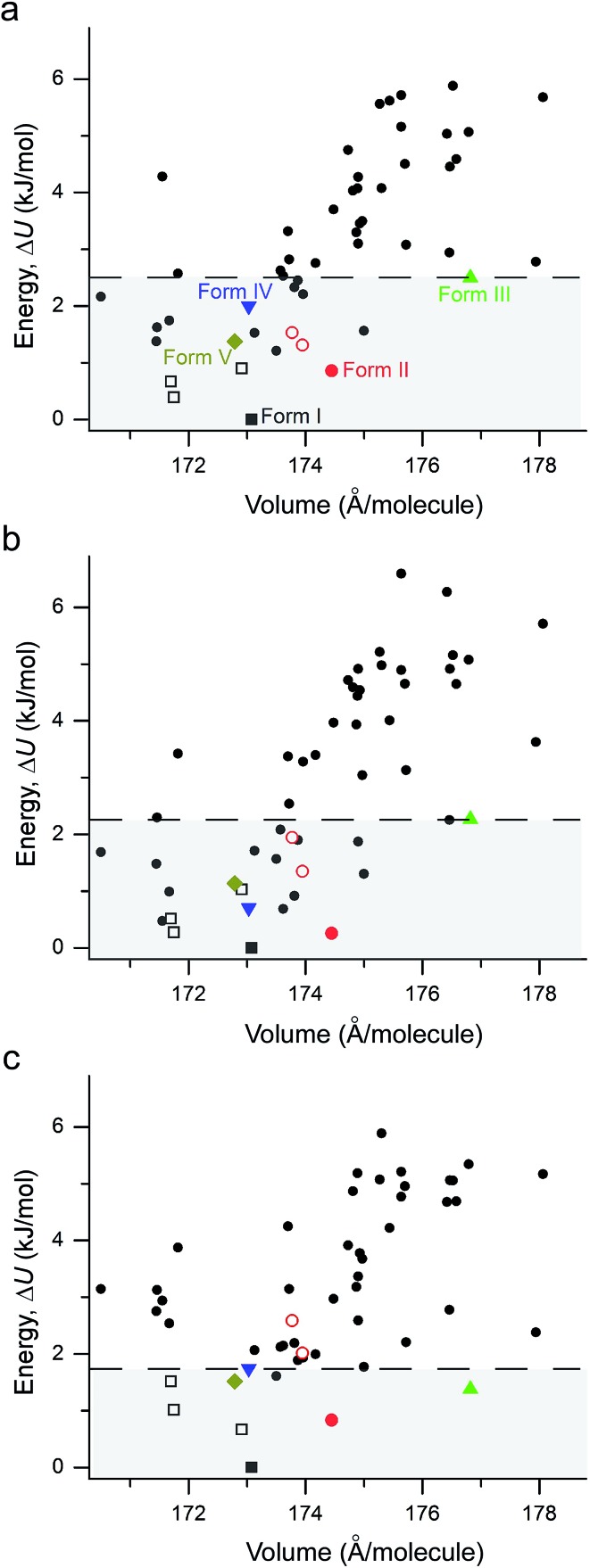
Lattice energy *versus* density plot for all low energy structures found in the present study. The structures are all optimized at the vdW-DF2 level with energies calculated with (a) vdW-DF2 functional implemented in Quantum ESPRESSO; (b) PBE+TS method in FHI-aims; (c) the PBE+MBD method in FHI-aims. **I** – black square, **II** – red circle, **III** – green up triangle, **IV** – blue down triangle, **V** – dark yellow diamond. The polytypic structures of **I** and **II** are marked with open symbols of the same colors and shapes. All experimental structures have energies within the range highlighted by the horizontal dashed line.

The performance of various vdW-inclusive methods has been recently reviewed^18,38^ and benchmarked on a range of systems.^52,53^ A study of the C21 reference set by some authors of this work has shown that accurate geometries and lattice energies can be obtained with the vdW-DF2 functional.^54^ Therefore, we used the vdW-DF2 functional implemented in the Quantum ESPRESSO code^55^ to relax 50 low-energy structures after merging results from CSP_A_ and CSP_B_. Not surprisingly, all observed metastable coumarin forms (namely, **II**, **III**, **IV**, and **V**) have very small energy differences relative to **I** (Fig. 9). However, it is well known that CSP methods generate more thermodynamically plausible structures than the number of known polymorphs.^56^ Indeed, several structures were generated by both CSP methods (see ESI† for a direct comparison), with a number of low energy structures within 5 kJ mol^–1^ of coumarin **I** sharing similar packing modes. For the 50 vdW-DF2 optimized structures (Fig. 9a), we also calculated energies using PBE+TS (Fig. 9b) and PBE+MBD (Fig. 9c) methods at the vdW-DF2 optimized structures with the all-electron code FHI-aims.^62^ With PBE+TS, a variety of structures have stabilities between the experimentally observed **I** and **V**. In contrast, in the PBE+MBD ranking, forms **I–V** are all observed within the 9 most stable structures, with 3 of 4 other experimentally non-observed structures being structurally very similar to **I** (see below). This remarkable energy separation between observed and non-observed structures already shows the importance of many-body interactions for the description of polymorph stabilities.

**Fig. 10:**
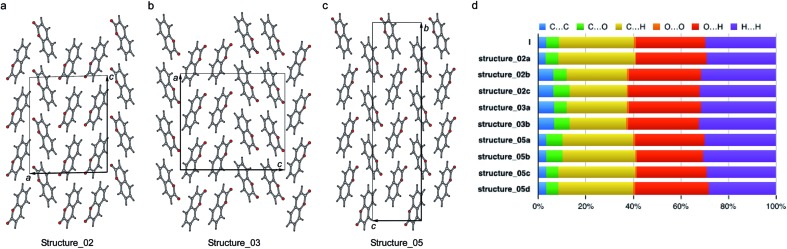
Three polytypic forms of **I** observed in CSP (which can be found in ESI† in a separate crystallographic information file referred as structure_02, structure_03, and structure_05, respectively).

In order to analyze the packing modes and intermolecular interactions, we use the fingerprint plots derived from Hirshfeld surfaces (Fig. 8 and 10 and ESI, Fig. S6†).^57,58^ We had previously used fingerprint plots in our study of pentamorphic 1,8-dihydroxyanthraquinone, another rare example of a multimorphic rigid molecule where we discovered three new polymorphs^59^ albeit single crystals from solution. The fingerprint plots of coumarin and 1,8-dihydroxyanthraquinone are surprisingly similar. In both cases (see ESI, Fig. S6† for coumarin), the lowest energy structure has “antennae” with internal (*d*_i_) and external (*d*_e_) distances of (1.4, 1.0 Å) and (1.0, 1.4 Å). This is indicative of C–H···O intermolecular distances which are shorter than the vdW distances (in this context, we consider them as weak hydrogen bonds). The “wings” of the fingerprint plots are due to C–H···π interactions. This combination is typical for herringbone structures such as these. The fingerprint plots of all the new polymorphs of coumarin are shown in ESI, Fig. S6,† and all of them contain a bright spot centered at *d*_i_ ∼ 1.9 Å, *d*_e_ ∼ 1.9 Å characteristic of π···π stacking, although the intensity does change. Again, this motif is found in three of the metastable polymorphs of 1,8-dihydroxyanthraquinone.

The percentage contributions of the close intermolecular contacts shown in Fig. 8 provide greater insight into the packing in the different polymorphs. All structures are dominated by C···H, O···H and H···H interactions. Although there are large changes in C···H and H···H across the series, the changes in O···H are smaller. Coumarin **I** has a relatively small C···C contribution and a correspondingly large C···H contribution, consistent with a herringbone type structure. In contrast, **II** is the opposite; it has a large C···C contribution expected for a structure with significant π···π stacking and a smaller C···H contribution. Coumarin **V**, the only other structure with *Z*′ = 1 has a breakdown that is intermediate between **I** and **II**. It does exhibit π···π stacking but the oblique angle of the molecules relative to each other in the crystal structure reduces the C···C interaction and provides for greater C···H contributions than in **I**. Both symmetry independent molecules in **III** have intermediate C···C contributions but quite different C···H contributions. The two symmetry independent molecules π stack with each other, leading to the C···C contribution. This dimer motif is then packed in such a way that there are no π···π interactions between dimers. The difference in C···H contributions is due to one molecule of the dimer interacting with O atoms around its edges, whereas the other interacts with H atoms. Coumarin **IV** is a mix of medium and high C···C interactions, a consequence each molecule *a* being sandwiched between a molecule of *b* and *c*, hence π-stacking with its two neighbours.

According to the lattice energy *versus* density plot at the level of PBE+MBD on top of the vdW-DF2 optimized structures (Fig. 9c), there are four other structures in the energy window of experimentally observed structures. Among these four, the one with highest energy is likely to be ruled out when a more accurate setting is applied. The remaining three structures are found to exhibit nearly identical 2D fingerprint plot patterns relative to **I** (Fig. S6†). Small differences arise in the contributions of the close intermolecular contacts shown in Fig. 10. Molecule *a* in structure_02 has a breakdown extremely similar to **I**. However, the other two molecules are different with a higher C···C contribution almost exclusively at the expense of the C···H contribution. Furthermore, the two molecules in structure_03 have almost identical breakdowns. This is evident in the fingerprint plots, where there is increased π···π stacking in two molecules in structure_03 and two molecules in structure_02. The difference between these molecules and the third molecule in structure_02 and molecules in **I** is clear in Fig. 10. Columns of molecules running along *c* doubled up in structure_02 and structure_03 lead to some π···π interactions, whereas the alternate columns in **I** do not. Finally, the four molecules in structure_05 all have contributions that are similar to **I**.

This analysis shows that all of these structures can be regarded as built up by stacking layers of the same units, differing only in the stacking sequence along various axes, similar to the stacking faults predicted for benzene at high pressure.^60^ Thus, they belong to the same polytypic family. We note that 30% of the low-energy structures predicted by both CSP methods were observed polymorphs or polytypes, providing an encouraging result for structure validation. From the calculated lattice energies, the energy penalty for alternative polytypes is quite small, but these structures have not been directly observed experimentally. These polytypes in coumarin may be kinetically unstable due to fast transformations to the more favorable known forms during crystal growth, making them unable to form large domains. The cocrystallization of low-energy polytypes may also play a role in the formation of twisted fibers in spherulites by creating long-range elastic stress fields.

## Energy ranking

**Fig. 11:**
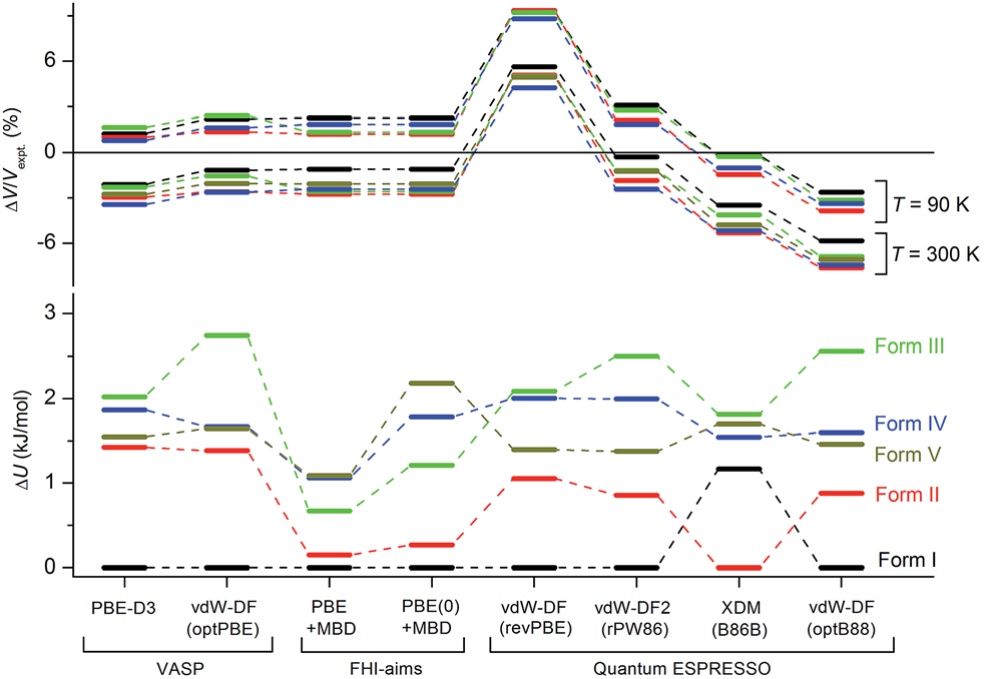
Comparison of different vdW-inclusive methods in terms of optimized unit cell volumes Δ*V*/*V*_expt_ in%, and energy rankings Δ*U* in kJ mol^–1^. Note that PBE+MBD and PBE(0)+MBD in FHI-aims used the relaxed geometry with light basis set at the level of PBE+MBD. For clarity, only eight vdW-inclusive methods are included in this figure. The overall comparison is shown in Fig. S7 in ESI.†

Although the C21 and X23 reference sets were carefully designed to cover a range of intermolecular interactions (*e.g.* H-bond, C–H stacking, *etc.*), only oxalic acid in this set shows polymorphism at ambient conditions. Therefore, it is questionable whether these reference sets are useful to benchmark energy rankings of vdW-inclusive methods in studies of crystal polymorphism. Indeed, we failed to reproduce the experimental stability ranking suggested by Table 1 and Fig. 4 for the new coumarin polymorphs when using vdW-DF2, as shown in Fig. 9a, although it was found to be one of the optimal choices in our earlier work.^54^ Hence we decided to use the observed set of coumarin polymorphs to test various popular dispersion models and correction schemes supported in various codes (including VASP,^61^ Quantum ESPRESSO,^55^ and FHI-aims^62^). These include empirical corrections (D2 and D3 without three-body dipolar interactions) combined with the PBE functional, two vdW-DF functionals and their optimized versions (optB88, optPBE), and also the TS and MBD model combined with PBE. We also estimated the impact of hybrid functionals by adding the energy difference between PBE0+MBD and PBE+MBD calculated at the light basis set in FHI-aims to the PBE+MBD energies obtained with the fully converged tight basis set using the PBE+MBD-optimized structures. This approach is labelled with PBE(0)+MBD. The benchmark results on cell volumes and lattice energies are shown in Fig. 11.

The original vdW-DF scheme was found to notably overestimate the unit cell volumes and this has been remedied by its later derivatives (vdW-DF2, optPBE, optB88).^19^ Here, we observed the same trend. All methods except vdW-DF underestimate the unit cell volumes by 1.18 to 9.63% compared to the room temperature data (Fig. 11). The unit cell volumes obtained at 90 K are about 3.61% smaller than the room temperature data set, and most of the vdW-inclusive methods have optimized structures within ±2% of the 90 K data. The description of the theoretical cell volumes could in principle be further improved by using the so-called quasi-harmonic approximation, which captures thermal-expansion effects, or by optimizing the unit cells with an appropriate thermal pressure.^41,63,64^ All methods give rather consistent differences (<3%) for the five polymorphs indicating that the small volume disagreement could be treated as a systematic error. Furthermore, given that related vdW-inclusive methods using different functionals give the same energy ranking with different relative energies and optimized cell volumes (see vdW-DF methods in Fig. S7†), we choose to focus our analysis on the energy rankings calculated using the DFT methods.

Unlike comparing predicted atomic positions with X-ray crystal structure coordinates, the comparison of calculated lattice energies is more challenging. Unfortunately, we are unable to obtain the sublimation enthalpies from the experiment, since all metastable coumarins convert to stable form **I**. However, the relative stabilities at room temperature can be derived according to the observed phase transformations (Fig. 4), namely, **I** > **II** > **III** > **IV** > **V**. Although it is difficult to check whether the sequence would change at temperatures approaching 0 K, we will assume that this ranking is independent of temperature for the following reasons, a collection of circumstantial evidence: (1) **I** is very likely the most stable form, since **I** was the only known form for a long time and there is no low temperature phase transition yet reported; (2) **III** and **IV** should be energetically close due to their structural similarity; (3) **V** is the least stable phase since it only remains observable for a short time under ambient conditions.

**Fig. 12:**
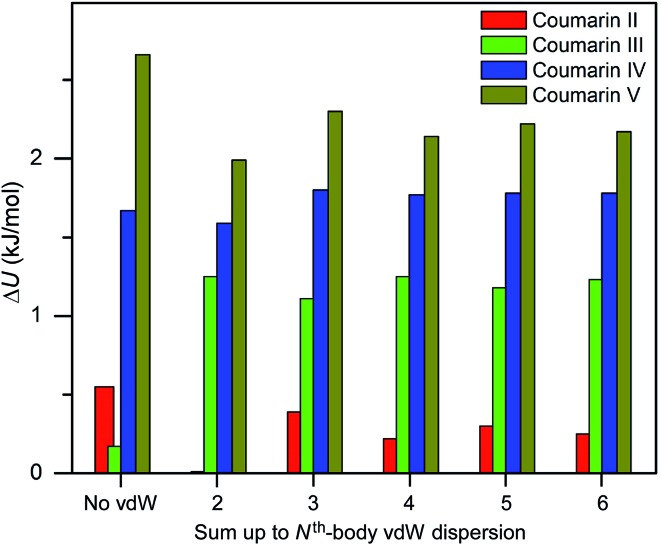
Comparison of lattice energy differences for all observed coumarin polymorphs relative to form **I** with different cutoffs of many body interactions within the framework of PBE(0)+MBD at the fully relaxed PBE+MBD structures *via* MBD code (Jan Hermann, source code of program MBD, Zenodo 2016, ; http://doi.org/10.5281/zenodo.47528).

Three methods (PBE-D2, XDM and PBE+TS) misrank **II** as the most stable form, while many methods identify **III** as the least stable form. Only three approaches using the MBD method (PBE+MBD in VASP and PBE/PBE(0)+MBD in FHI-aims), yield the results satisfying the above criteria, and coincidentally produce the same stability ranking as observed at room temperature (**I** > **II** > **III** > **IV** > **V**), despite the fact that magnitudes differ by 1 to 2 kJ mol^–1^ due to the choices of codes and functionals. This also agrees with our finding that the PBE+MBD model yields the best energy separation between observed and non-observed structures predicted by CSP. Although both PBE-D3 and XDM-B86B were found to have a similar level of accuracy as PBE(0)+MBD for X23 in a recent review,^18^ they clearly fail in the case of coumarin polymorphs. A possible explanation might be that these models fail to take into account the many-body interactions. Fig. 12 shows the lattice energy ranking notably changes by including the many-body contributions from pairwise up to 6^th^ order within the MBD model, in which the term body refers to individual atoms. It can be seen that in this case, 3-body contributions are crucial for determining the relative stability ordering and higher-order contributions still modify the relative energies by up to 0.2 kJ mol^–1^. This analysis only shows the effect on the lattice energy but not for the geometry or vibrational free energies. The importance of many-body dispersion effects for energies and for response properties is discussed in a recent review.^63^ It has been found that MBD plays an essential role in the stability rankings on various systems such as aspirin^65^ and glycine.^66^ Our results suggest that coumarin crystals also exhibit strong many-body interactions, and this could serve as a supplementary data set to validate different vdW-inclusive models in addition to the widely used X23 set.

## Free energy under finite temperature

In studies of organic crystals, the free energy is usually approximated as the static lattice energy due to computational limitations. However, recent studies have shown that the addition of vibrational free energy contributions affects polymorph stability rankings.^65,67^ In order to explore these contributions, we calculated the vibrational free energies for all coumarin polymorphs in a harmonic fashion using a finite displacement approach. The stability ranking is obtained by adding the respective harmonic vibrational free energy (calculated for the PBE+MBD structures at 0 K) to the static lattice energy obtained with PBE(0)+MBD. The relative stabilities as a function of temperature are plotted in ESI, Fig. S8.† At 300 K, the free energy ranking changes from **I** < **II** < **III** < **IV** < **V** (the expected order) to **I** < **V** < **II** < **IV** ≈ **III** (Table 4). Therefore, the PBE(0)+MBD free energies satisfy two out of the three previously mentioned experimental stability observations, but **V** is significantly stabilized when harmonic vibrations and zero-point energies are included. Form **V** is the second most stable polymorph even when a larger basis set or the experimentally-obtained lattice constants at 300 K are used (see ESI, Table S3†). These results suggest that anharmonic effects probably play an important role at or above room temperature, as seen in the case of paracetamol,^68^ and should be expected to become more pronounced near the melting point of coumarin (around 340 K). However, the calculation of accurate anharmonic free energies on a fully first-principles level for all polymorphs of coumarin is far beyond the available computing resources.

**Table 4:** Lattice energy (Δ*E* at 0 K) or free energy (Δ*G* at 300 K) difference relative to coumarin **I** in kJ mol^–1^ ^*a*^

	PBE(0)+MBD		OPLS		
Polymorph	Δ*E*	Δ*G* (harmonic)	Δ*E*	Δ*G* (harmonic)	Δ*G* (anharmonic)
Coumarin **II**	0.27	0.70	1.58	2.02	4.5 ± 0.7
Coumarin **III**	1.21	0.94	4.17	2.73	n/d
Coumarin **IV**	1.78	0.90	3.72	1.47	n/d
Coumarin **V**	2.18	0.17	5.47	4.22	16.0 ± 1.6

^*a*^“n/d” – not determined.

Therefore, we returned to the modified OPLS force field to further investigate thermal effects using classical MD. The classical force field energy ranking of optimized structures is **I** < **II** < **IV** < **III** < **V**. A comparison of relative energies over the full range of predicted structures shows that the DFT energies are typically only 60% of the OPLS-based energies (see ESI†), hence, an overestimate of the relative energies for observed polymorphs is expected (see Table 4). To calculate the relative free energies we first used the harmonic approximation as described above. Adding the vibrational free energy contribution to the fully optimized (0 K) structures, form **IV** becomes the second most stable polymorph above 250 K. The harmonic approximation of free energies shows the same trends as for PBE(0)+MBD, with the energy gap relative to form **I** decreasing at higher temperatures for all observed structures other than **II**. If the average cell vectors from the MD simulations at 300 K are used to account for thermal expansion, the energy differences relative to form **I** are reduced, with a free energy ranking of **I** < **IV** < **III** < **II** < **V** (see ESI, Fig. S9†). With the exception of form **V**, this ranking also agrees with the DFT results using experimental lattice vectors, suggesting that the classical force field can be used to obtain appropriate rankings with overestimated relative energies. However, both methods result in relative free energies at 300 K that are considerably larger than the estimates based on the heat of fusion for form **I** at the melting temperature (Table 1).

To further evaluate the free energy differences between structures at temperatures near the melting point, we extend the classical analysis to allow for anharmonic effects in the MD simulations. Although this could be done using λ-path integration from a harmonic or quasi-harmonic reference to a fully an harmonic description, as recently reviewed by Moustafa *et al.*,^78^ we chose instead, as in our previous studies^69,70^ to use thermodynamic integration to compute the free energy difference between polymorphs based on a given path between structures. Using steered MD simulations (see ESI† for details of the collective variables used for each supercell), the relative free energy of forms **I**, **II**, and **V** were calculated along paths that interconvert these structures. Even with classical MD, the computational cost of this approach limited the analysis to the polymorphs with 4 or fewer molecules in the unit cell.

The relative energy and free energy rankings for the coumarin polymorphs are summarized in Table 4. Importantly, these calculations show that including the vibrational free energy contribution changes the energy ranking of coumarin polymorphs for both DFT and classical force field methods, particularly when the thermally expanded lattice vectors are used (see ESI†). All structures other than form **II** have the same trend in relative free energy and become more likely at higher temperatures, consistent with the newly characterized polymorphs being crystallized from the melt. However, the fully anharmonic calculations show an even greater change in the relative free energies of polymorphs **II** and **V**. Even though the classical polymorph relative lattice energies are known to be overestimated, this result suggests that non-negligible contributions from anharmonic vibrations must be included to properly rank the stabilities of coumarin polymorphs at temperatures above 100 K, despite the considerable computational cost.

## Conclusions

The preparation of five polymorphs of coumarin, a simple, rigid, and well-characterized compound, was only possible by crystallization from the melt, a technique less commonly used in polymorph screening. Since the samples were polycrystalline, we used powder X-ray diffraction methods to obtain structural information. To solve the crystal structures, we relied on crystal structure prediction, a set of techniques that are becoming more suitable to a wider range of systems.^13^ Solution of crystal structures from PXRD data using CSP methods is not common but it is a promising strategy well illustrated by coumarin. A recent study has shown that multiple independent molecules greatly complicate traditional crystal structure search based on quasi random sampling,^71^ despite a few successful studies reported in the literature.^72,73^ Our success in solving coumarin **IV** in the present study is encouraging and suggests that the efficiency can be greatly enhanced by the advanced global optimization methods such as the evolutionary algorithm USPEX used here towards solving crystal structures with *Z*′ > 2.

Another challenge of CSP techniques is that the ranking of predicted structures is based on calculated energies. Despite the fact that many vdW-inclusive methods have been proposed and more are under active development, our benchmark calculations on coumarin suggest that only a few models produce good agreement with experimental results. In particular, inclusion of many-body dispersion interactions is crucial for the stability ranking. Computation of harmonic free energies is used increasingly for polymorph ranking.^13^ However, the results for coumarin suggest that for some stability trends, harmonic free energies are not sufficient and anharmonic effects must be considered as well.

## Experimental

A few mg of coumarin (Sigma-Aldrich, 99%) mixed with 0–40 wt% Canada balsam (if the concentration of Canada balsam is not stated it was 21 wt%) were placed between a microscope slide and a glass cover slip and melted on a Kofler bench at *ca*. 75 °C. Then the samples were cooled and crystallized either at room temperature or on a Kofler bench at 30–50 °C or in a refrigerator at 4 °C. Some samples were re-melted and subsequently crystallized on a hot stage (Model FP90, Mettler-Toledo) at 30–69 °C. Polarized light micrographs were made with an Olympus BX50 microscope equipped with a digital camera.

X-ray diffraction (XRD) patterns were collected using a Bruker AXS D8 DISCOVER GADDS microdiffractometer equipped with a VÅNTEC-2000 two-dimensional detector and a 0.5 mm MONOCAP collimator (Cu Kα radiation, step size 0.01°). The data collection was performed in reflection mode either from an as-grown crystalline film on a glass slide with the cover glass removed or from a powder detached from the glass slide and attached to a silicon wafer with a small amount of vacuum grease.

High-resolution synchrotron powder diffraction data were collected at the ID22 beamline of the ESRF at a wavelength of 0.41064(1) or 0.39992(1) Å, step size 0.002°. The powder of coumarin was detached from the glass slide and placed into 1 mm borosilicate glass capillary. The patterns were collected immediately afterwards at room temperature and at 90 K using a cryostream.

Raman spectra were collected with a Thermo Scientific DXR Raman microscope (laser wavelength 532 nm, laser power 4 mW) from an as-grown crystalline film on a glass slide covered with cover glass (coumarin **V**) or with the cover glass removed (coumarin **I**, **II**, **III**, and **IV**).

The melting point and the heat of fusion were measured using a Perkin-Elmer DSC 8000 differential scanning colorimeter (DSC) for ∼5 mg sample of coumarin sealed in a hermetic aluminium pan.

## Computational details

### Force field and structure generation

For CSP_B_, the standard OPLS force field^37^ was modified to use ESP-fitted atomic charges based on the electron density from a DFT-optimized single molecule (PBE0/6-311G* in Gaussian09).^27,35,36^ In the UPACK^34^ random search, lattice energies were evaluated using a cutoff of 12 Å with an Ewald damping range of *α* = 3 nm^–1^ and reciprocal space cutoff of 2 nm^–1^ for both Coulomb and dispersion terms. These structures were clustered with the radial distribution function available in UPACK, using a cutoff of 7 Å and a tolerance of 0.25 Å to remove duplicates.

### MD simulations

The 20 lowest energy structures from the random structure CSP_B_ and the 4 observed polymorphs were passed through a flexible-cell NPT molecular dynamics screening to evaluate the stability of each packing motif. MD simulations were run using the PINY_MD package^74^ with details of the runtime parameters reported in the ESI.† After equilibration of at least 100 ps, a window of 50–100 ps was used as the production run to obtain averaged unit cells and lattice energies.

### Energy ranking

The 100 lowest energy structures from CSP_A_ and 15 lowest energy structures from CSP_B_ were re-optimized using the vdW-DF2 functional as implemented in Quantum ESPRESSO using the projector-augmented wave (PAW) method.^75^ A plane wave kinetic energy cutoff of 80 Ry was used, and pseudo potentials were adapted from the atompaw library.^76^ Among the total 115 structures, we chose the 50 lowest energy structures for further analysis after removing the duplicates. The crystallographic information for the 50 lowest energy structures is also deposited in the ESI.†

For the relative energy ranking of the experimentally observed forms, we optimized the structures using various vdW-inclusive methods available in Quantum ESPRESSO, VASP and FHI-aims. For Quantum ESPRESSO, the same parameter set as described in the previous section is used. In VASP, the plane-wave kinetic energy cutoff used is 1000 eV. For FHI-aims, light species default settings were used for lattice and geometry optimizations, while tight species default settings were used for the final energy calculations. For all geometry relaxation calculations, the Brillouin zone was sampled by uniform *Г*-centered meshes with the reciprocal space resolution at least 2π × 0.06 Å, with convergence criteria of 1 × 10^–5^ eV per atom for total energies, 5 × 10^–3^ eV Å^–1^ for forces.

### Phonon calculations

Phonon calculations were performed for structures **I–V** in the finite displacement approach within the harmonic approximation by using the all-electron DFT code FHI-aims and Phonopy.^77^ All forces were calculated at the PBE+MBD level of theory using light settings in FHI-aims. In order to avoid artefacts, supercells with a length of at least 10 Å in each cartesian direction have been used.

### Classical harmonic approximation

To calculate the free energy using the harmonic approximation and the OPLS-based force field, the entropy contribution was determined by considering atomic vibrations as a system of non-interacting harmonic oscillators, with frequencies given by the eigenvalues of the Hessian matrix. Unit cell vectors for each polymorph were determined by averaging 100 ps isothermal–isobaric (NPT_F) MD trajectories for a range of temperatures. Atomic positions were then optimized within each fixed unit cell before computing the Hessian matrix using the finite displacement method with a repeating unit cell at least 12 Å in each dimension.

### Classical thermodynamic integration

Using a set of collective variables (CVs), we implemented steered MD to interconvert coumarin phase **I**, **II**, and **V** by assigning molecular equivalencies within a small supercell (see ESI† for details). Then we applied thermodynamic integration based on the supercell matrix (whose columns are the supercell vectors) and the respective CVs, obtaining the relative free energy difference^69^ at 100, 200, and 300 K. The CVs used were based on the distance between molecular centers of mass and relative molecular quaternions. A more detailed discussion can be found in the ESI.†
