# Supplementary material for: Powder diffraction and crystal structure prediction identify four new coumarin polymorphs
Source: Chem Sci. 2017 May 15;8(7):4926–40. doi: 10.1039/c7sc00168a (PMC5607859; doi:10.1039/c7sc00168a)
Supplement: Supplementary file 1 [file SC-008-C7SC00168A-s001.pdf]

Electronic Supplementary Material (ESI) for Chemical Science.
This journal is © The Royal Society of Chemistry 2017

Powder Diffraction and Crystal Structure Prediction Identify Four New Coumarin Polymorphs

Alexander G. Shtukenberg,[a*] Qiang Zhu,[bc*] Damien J. Carter,[d] Leslie Vogt,[e] Johannes Hoja,[fg] Elia Schneider,[e] Hongxing Song,[e] Boaz Pokroy,[h] Iryna Polishchuk,[h] Alexandre Tkatchenko,[fg] Artem R. Oganov,[ic] Andrew L. Rohl,[d] Mark E. Tuckerman[ejk] and Bart Kahr[al]

[a]Department of Chemistry and Molecular Design Institute, New York University, New York City, NY, 10003, USA

[b]Department of Physics and Astronomy and High Pressure Science and Engineering Center, University of Nevada Las Vegas, Nevada 89154, USA

[c]Department of Geosciences, Stony Brook University, Stony Brook, NY, 11794, USA

[d]Curtin Institute for Computation and the Department of Chemistry, Curtin University, P.O. Box U1987, Perth, 6845, Western Australia, Australia

[e]Department of Chemistry, New York University, New York City, NY, 10003, USA

[f]Fritz-Haber-Institut der Max-Planck-Gesellschaft, Faradayweg 4-6, Berlin, 14195, Germany

[g]Physics and Materials Science Research Unit, University of Luxembourg, 1511 Luxembourg, Luxembourg

[h]Department of Materials Science and Engineering and the Russell Berrie Nanotechnology Institute, Technion Israel Institute of Technology, Haifa 32000, Israel

[i]Skolkovo Institute of Science and Technology, Skolkovo Innovation Center, 3 Nobel St., Moscow 143026, Russia

[j]Courant Institute of Mathematical Science, New York University, New York City, NY, 10003, USA

[k]New York University-East China Normal University Center for Computational Chemistry at NYU Shanghai, 3663 Zhongshan Road North, Shanghai 200062, China

[l]Department of Advanced Science and Engineering (TWIns), Waseda University, 162-0056 Tokyo, Shinjuku, Wakamatsucho, 3−2, Japan

Table of Contents

I. Structural Data

Figure S1. Diffraction pattern of **III** at room temperature.

Figure S2. Diffraction pattern of **IV** at room temperature.

Table S1. Comparison of structures of coumarin polymorphs.

II. Comparison of Crystal Structure Prediction Methods

Table S2. OPLS parameters.

Figure S3. Number of CSP-generated structures

Figure S4. Comparison of relative energies of CSP structures

Figure S5. Comparison of relative energies of CSP structures

III. Hirshfeld surfaces

Figure S6. Hirshfeld surfaces of five experimental coumarin polymorphs and three predicted coumarin structures corresponding to polytypes of **I**

IV. Comparison of different DFT-based vdW-inclusive methods

Figure S7. Comparison of different vdW-inclusive methods.

V. Free Energy Comparison

Figure S8. PBE(0)+MBD harmonic free energies versus temperature of all experimentally observed polymorphs relative to form **I**.

Table S3. PBE(0)+MBD free energy (ΔG) differences at 300 K relative to coumarin I in kJ mol^{-1} calculated at the fully-relaxed structures with PBE+MBD phonons obtained with the light basis (light phonons) and tight basis (tight phonons) in FHI-aims, and calculated at the experimental lattice constants corresponding to room temperature with PBE+MBD phonons obtained with light basis (expt. lattice). "n/d" indicates values that were not determined.

Figure S9. Relative energy of coumarin polymorphs using the classical harmonic free energy at temperatures from 50 – 350 K.

Figure S10. Simulated supercells for steered MD.

Table S4. Relative free energy differences for select coumarin polymorphs based on steered MD (kJ mol^{-1})

Additional Files:

CIF file of 50 structures from Crystal Structure Prediction

CSP_structures.cif and CSP_structures.txt

CIF file combining all experimental structures

Coumarin **II** (room temperature); **III** (T = 90 K and room temperature); **IV** (T = 90 K and room temperature); **V** (room temperature).

I. Structural Data

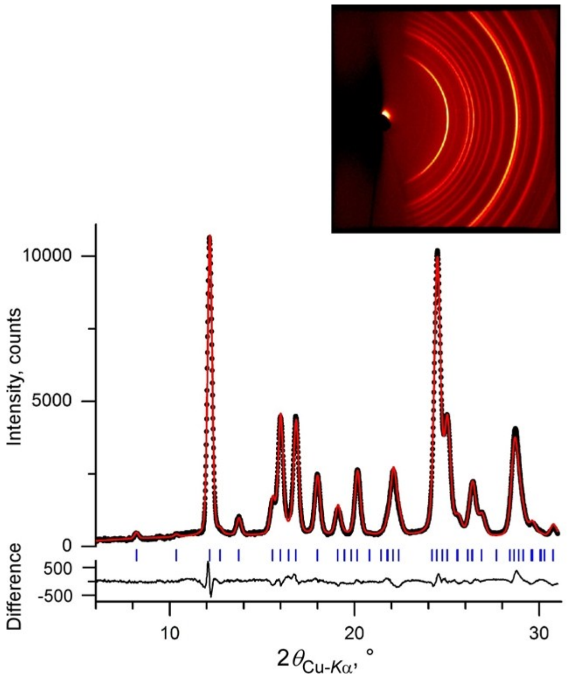

Fig. S1 Room temperature 2D diffraction pattern (inset) and corresponding integrated intensities of a powder sample of **III** (black dots). Sample contains 21 wt.% Canada balsam. Red line corresponds to the simulated diffraction pattern. The data were collected with D8 DISCOVER GADDS microdiffractometer at room temperature. Blue ticks are reflection positions. The lower trace shows the difference curve.

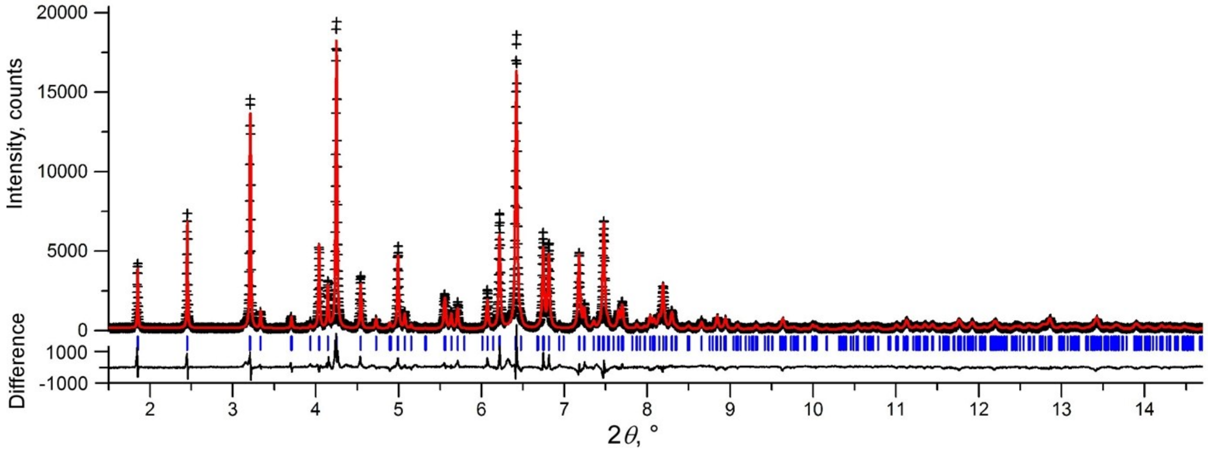

Fig. S2 Rietveld refinement of high-resolution synchrotron powder diffraction for **IV** sample containing 21 wt.% Canada Balsam. Observed (black crosses) and calculated (red line). The data were collected at the ESRF at a wavelength of 0.39992(1) Å and at room temperature. Blue ticks are reflection positions. The lower trace shows the difference curve.

Table S1 Comparison of structures of coumarin polymorphs (data collected at 90 K)

Polymorph	Coumarin **I**[a]	Coumarin **II**[b]	Coumarin **III**[b]	Coumarin **IV**[b]
Space group	$Pca2_1$	$P2_1$	$P2_12_12_1$	$P2_12_12_1$
a (Å)	15.478(4)	3.852(1)	16.782	24.270
b (Å)	5.6091(13)	15.284(1)	5.921	5.921
c (Å)	7.7343(19)	5.813(1)	13.852	14.189
β (°)	90	86.41(3)	90	90
V (Å^3)	671.5	341.6	1376.4	2039.0
Z, Z'	4, 1	2, 1	8, 2	12, 3

[a]Data are taken from P. Munshi and T. N. G. Row, *J. Phys. Chem. A*, 2005, **109**, 659. [b]Data collected at ESRF. If the errors of lattice parameters from least squares fitting are not shown they are too small (1-2 × 10^{-4} Å) to be physically meaningful.

II. Comparison of Crystal Structure Prediction Methods

This work used two independent crystal structure prediction (CSP) methods to understand which forms of coumarin are obtained from the melt. Comparing the low-energy structures generated by both CSP approaches provided an internal check on the proposed crystal structures (and polytypes) and validated the OPLS-based force field for use in molecular dynamics simulations of coumarin (see SI Section V).

CSP_A used a two-step approach to determine possible coumarin crystal structures. In the first step, the rigid-molecule structure from the previously reported experimental form (CSD entry: COUMAR12) was used to generate possible crystal structures in the USPEX code.[1,2] Each candidate structure was optimized using rigid-molecule relaxation as implemented in DMACRYS,[3] with FIT[4] empirical repulsion-dispersion potentials and a distributed multipole model[5] constructed from the Møller-Plesset MP2/6-31G(d,p) charge density calculated at the experimental geometry in Gaussian09.[6] The full search used multiple CSP runs using the 30 most common space groups for organic molecules for Z'=1 and 2, and $P2_1/c$, $P2_12_12$, $P2_12_12_1$, $Pca2_1$, and $Pna2_1$ for Z'=3 and 4. For each search in the context of the evolutionary algorithm, the population size is typically set to 100 and runs were terminated after 50 generations for Z'=1 and 2, or 100 generations for Z'=3 and 4. To rank the structures as the second step in CSP_A, the 100 lowest-energy structures for each search were fully optimized with periodic DFT in VASP [7] using the vdW-DF(optPBE) functional with a plane wave cutoff of 1000 eV. The final CSP_A ranking was based on these energies. After merging the 100 structures from each CSP run and removing duplicates, the 50 lowest-energy structures were selected for further analysis in this work.

In the CSP_B approach, structure generation, optimization, and ranking were performed at the same time. For this CSP search, the rigid-molecule structure generation used a DFT-optimized coumarin molecule (PBE0/6-311G* in Gaussian09)[6,8,9] in a UPACK[10] random search with an external pressure of 1 bar. For energy evaluations, the standard OPLS force field[11] was modified to use ESP-fitted atomic charges for the electron density from the DFT optimization. Charges and atom types are shown in Scheme S1 and non-bonded parameters are given in Table S2. Lattice energies were evaluated using a cutoff of 12 Å with an Ewald damping range of $\alpha = 3\ nm^{-1}$ and reciprocal space cutoff of $2\ nm^{-1}$ for both Coulomb and dispersion terms. These structures were clustered with the radial distribution function available in UPACK, using a cutoff of 7 Å and a tolerance of 0.25 Å to remove duplicates.

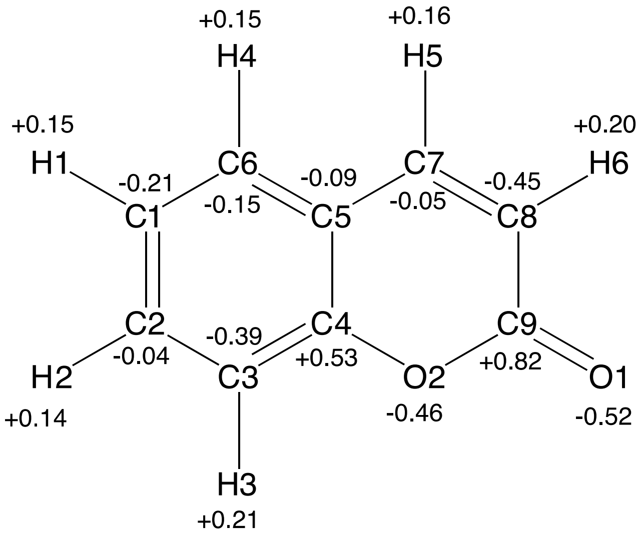

Scheme S1 Charge assignments and atom types for coumarin in CSP_B.

Table S2 OPLS non-bonded parameters[11] for CSP_B.

Atom Type	σ (Å)	ε (kcal mol^{-1})
C1-C8	3.55	0.070
C9	3.75	0.105
O1	2.96	0.210
O2	3.00	0.170
H1-H6	2.42	0.030

The initial search involved generating 1000 structures in each of 13 common space groups ($P2_1/c$, P-1, $P2_12_12_1$, $P2_1$, *Pbca*, *C2/c*, $Pna2_1$, *Cc*, $Pca2_1$, *C*2, *P*1, *Pbcn*, *Pc*) for *Z'*=1 and 2. After clustering, 58 unique structures were within 5 kJ/mol of the lowest energy packing, which is coumarin **I**. (In comparison, CSP_A has 26 *Z'*=1 or 2 structures within 5 kJ mol^{-1}

using vdW-DF(optPBE) in VASP.) To further investigate packing motifs, a subsequent search was run for each of the 13 space groups with 4000 additional structures using *Z'* = 1 and 2, and 5000 structures for Z' = 3. Clustering all generated structures resulted in a total of 104 unique structures within 5 kJ mol^{-1} of **I**. As this set of structures did not contain coumarin **IV,** a dedicated search with *Z'* = 3 in the $P2_12_12_1$ space group found that this packing conformation was generated only once in 60,000 structures; this sampling problem highlights the challenge of CSP for larger numbers of molecules in the asymmetric unit.

The set of structures generated by CSP_A and CSP_B were compared using clusters of 20 molecules in the COMPACK algorithm[12] as implemented in Mercury 3.8. Any pair of structures with an $RMSD_{20}$ < 0.6 Å was inspected by hand to ensure that the packing was identical. This step was necessary due to false matches recorded between *Z'* = 3 or 4 structures from CSP_A matching *Z'* = 1 or 2 structures from CSP_B. Many of these false matches were structurally identical for the local region, but represented different polytypes of coumarin in the larger unit cell. For example, the *Z'* = 4 structure highlighted Fig. 10c was identified as a local match for three CSP_B structures. Some higher energy *Z'* = 3 structures were paired with up to six different CSP_B unit cells.

The two CSP methods use different generation and ranking approaches, but the comparison of low-energy structures indicates that many of these were found by both methods. The comparison of unique generated structures with lattice energies within 5 kJ/mol of coumarin **I** is shown in Fig. S3. Although each CSP method samples a different set of structures, the 16 common structures in this energy window include experimentally observed polymorphs **I** and **II** and related polytypes. In an expanded energy range of 7 kJ/mol (as seen in Fig. S4), 8 of the 29 structures found by both CSP_A and CSP_B are the observed polymorphs or related polytypes. The result of the comparison confirms that the observed structures reported in this work are found regardless of structure generation approach.

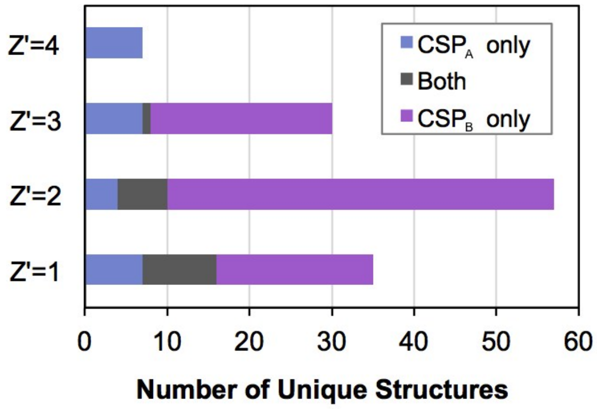

Fig. S3 Distribution of unique structures generated by CSP methods using an evolutionary algorithm (CSP_A within 5 kJ mol^{-1} of **I** according to vdW-DF(optPBE) in VASP) and a random search (CSP_B within 5 kJ mol^{-1} of **I** according to OPLS-based force field).

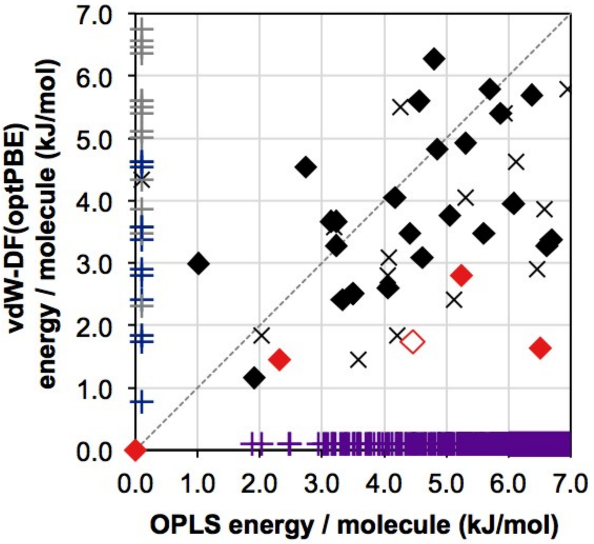

Fig. S4 Comparison of lattice energies for low-energy structures optimized from CSP_A using vdW-DF(optPBE) in VASP and structures generated using OPLS-based CSP_B. Red diamonds indicate the observed polymorphs, with an open symbol showing the OPLS-based energy of **IV** determined after the structure was known. Black diamonds indicate the energies of 25 other structures found by both methods and × symbols denote false matches (see text). Crosses along the *x*-axis show energies of 460 unique structures found only by CSP_B while crosses along the *y*-axis show energies for 9 structures for *Z*'=1,2,3 (blue) and 17 structures for *Z*'=4 (grey) found only by CSP_A.

The structural matches between CSP_A and CSP_B have heavy atom $RMSD_{20}$ values < 0.6 Å (most structures have $RMSD_{20}$ < 0.3 Å). The rigid coumarin molecule used in CSP_B (optimized with DFT in gas phase) has $RMSD_1$ < 0.06 Å when compared to any single molecule from CSP_A structures (optimized with DFT in crystal environments). These results show that the deviations in CSP energy evaluations are not due to structural differences.

To further evaluate the energy evaluation error of the classical, fixed charge OPLS-based force field, the set of 50 low-energy structures evaluated using DFT (vdW-DF2 in Quantum ESPRESSO) was used to quantify the error in relative lattice energies. In general, the DFT relative energies are ~ 60% of those calculated using the OPLS-based force field. The mean unsigned error (MUE) for the OPLS-based force field relative lattice energy is 1.8 kJ mol^{-1} (Figure S5). For observed structures, the MUE is 2.9 kJ mol^{-1}, which indicates that the polarization of coumarin molecules in the observed crystalline phases is important to the stabilization of these polymorphs. This also serves as a cautionary example of the increased

energy range that must be considered when using a non-polarizable force field when generating possible structures.

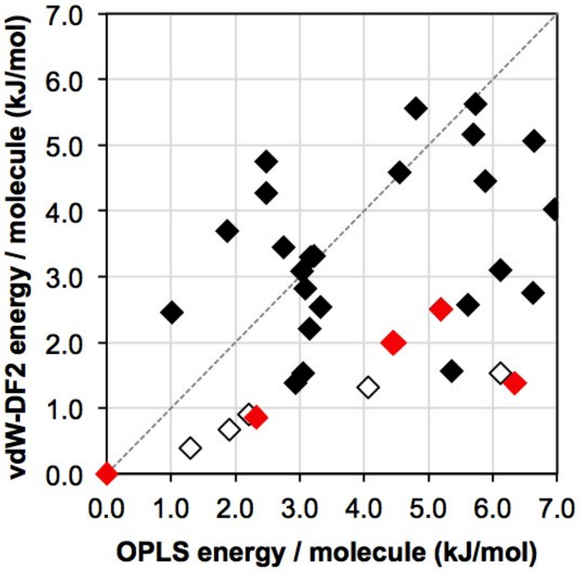

Fig. S5 Comparison of lattice energies relative to coumarin **I** for low-energy structures using the OPLS-based force field and vdW-DF2 in Quantum ESPRESSO. Diamonds indicate the observed coumarin polymorphs (red) and all other structural matches (black), with open symbols showing polytypes of the observed structures.

III. Hirshfeld surfaces

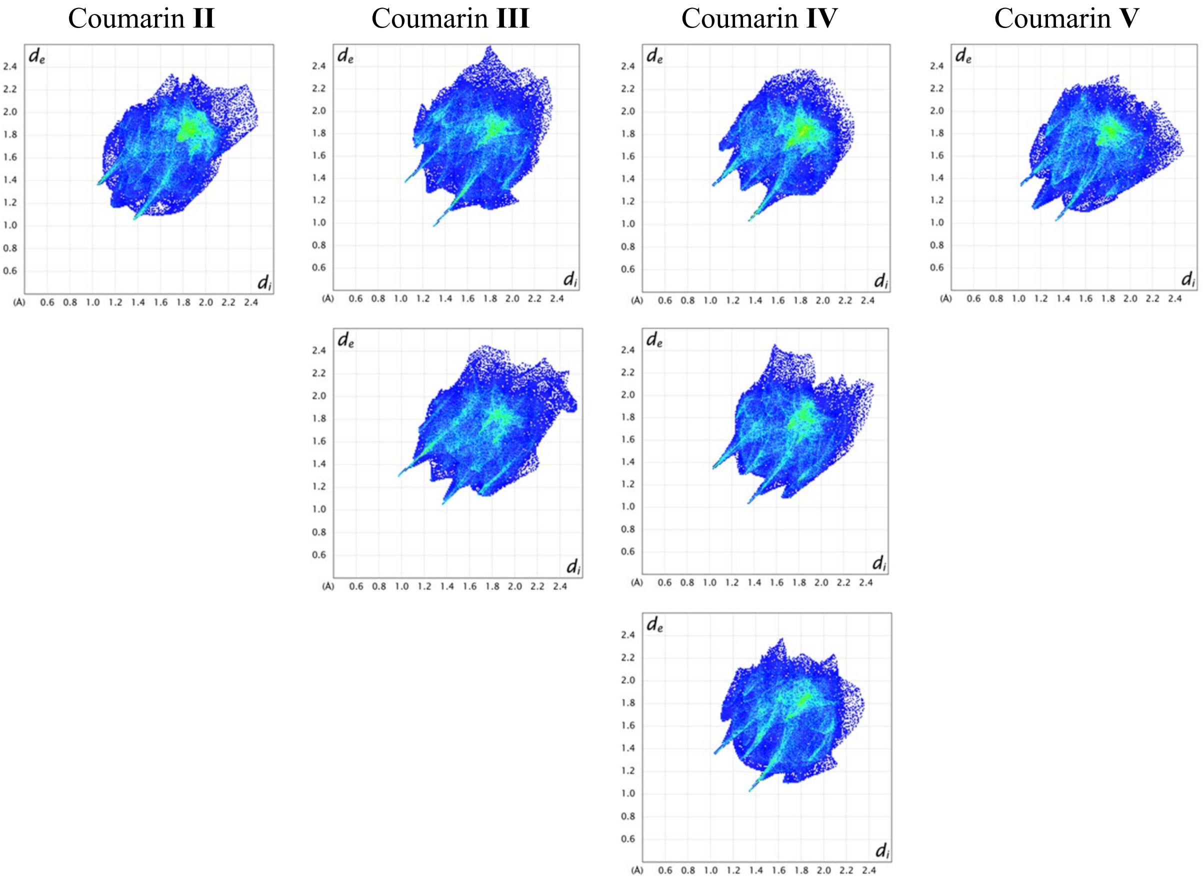

Fig. S6 Hirshfeld surfaces of four experimental coumarin polymorphs (see Fig. 8).

Coumarin **I** | Structure_02 | Structure_03 | Structure_05

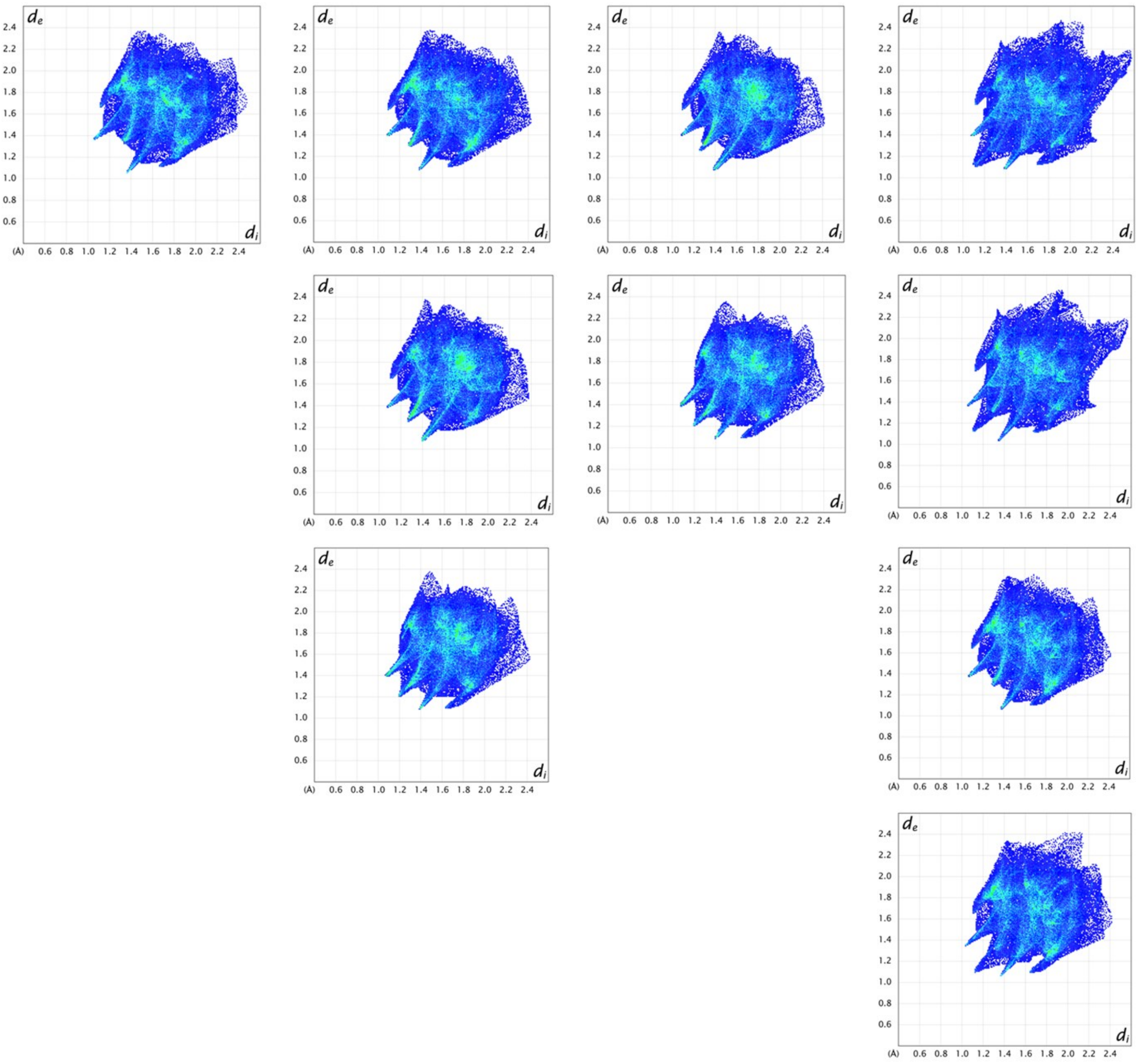

Fig. S6 (continued) Hirshfeld surfaces of experimental coumarin **I** and three predicted coumarin structures corresponding to polytypes of **I** (see Fig. 8, 10).

IV. Comparison of different DFT-based vdW-inclusive methods

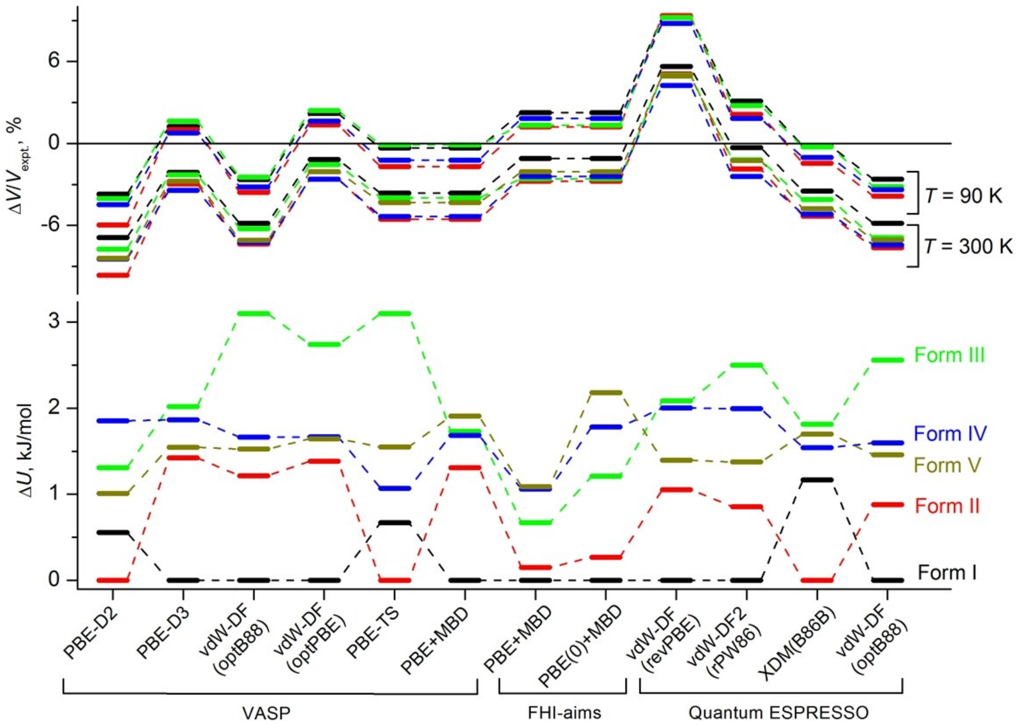

Fig. S7 Comparison of different vdW-inclusive methods in terms of optimized unit cell volumes $\Delta V/V_{expt}$ in %, and energy rankings ΔU in kJ mol^{-1}. Note that the PBE+MBD energies in VASP were calculated on top of the PBE+TS optimized geometries, while both PBE+MBD and PBE(0)+MBD in FHI-aims used the relaxed geometry with light basis set at the level of PBE+MBD.

V. Free Energy Comparisons

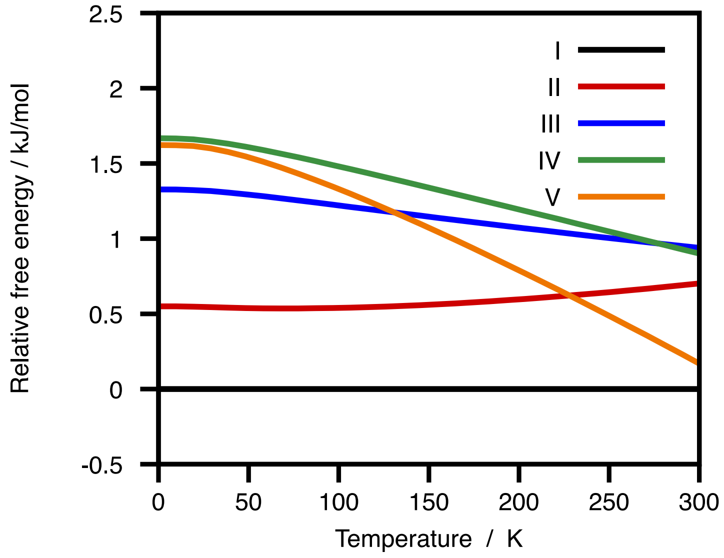

Fig. S8 PBE(0)+MBD harmonic free energies vs. temperature of all experimentally observed polymorphs relative to form **I**.

Table S3 PBE(0)+MBD free energy (ΔG) differences at 300 K relative to coumarin **I** in kJ mol^{-1} calculated at the fully-relaxed structures with PBE+MBD phonons obtained with the light basis (light phonons) and tight basis (tight phonons) in FHI-aims, and calculated at the experimental lattice constants corresponding to room temperature with PBE+MBD phonons obtained with light basis (expt. lattice). "n/d" indicates values that were not determined.

polymorph	light phonons	tight phonons	expt. lattice
Coumarin **II**	0.70	n/d	1.82
Coumarin **III**	0.94	n/d	1.43
Coumarin **IV**	0.90	n/d	1.30
Coumarin **V**	0.17	0.30	0.26

Classical Molecular Dynamics

To evaluate the stability of packing motifs generated by the CSP_B approach, the five observed polymorphs and 20 other low-energy structures were passed through a flexible-cell isothermal-isobaric NPT molecular dynamics (MD) screening with flexible coumarin molecules. MD simulations were run using the PINY_MD package[13] with massive Nosé–Hoover chain (NHC) thermostats[14] for atomic degrees of freedom (length = 2, τ = 20 fs, Suzuki–Yoshida order = 7, multiple time step = 4) and a time step of 1 fs. The pressure was kept at P = 1 bar, using the MTK barostat[15,16] and reversible, measure-preserving integrator of Tuckerman and coworkers[17] (τ = 1000 fs) and an NHC thermostat (τ = 1000 fs) on the barostat. After equilibration of at least 100 ps, the following 100 ps were used as the production run to obtain averaged unit cells and lattice energies. All structures examined were observed to be local minima, although MD-averaged cell vectors were larger due to thermal expansion (< 5% change in all cases).

Using the averaged unit cells obtained for the observed polymorphs at a series of temperatures from 50 to 350 K, the atomic positions were optimized. The Hessian matrix for each cell is calculated numerically by displacing each atom in the average unit cell to obtain the vibrational contribution to the free energy. The relative free energies as a function of temperature (Fig. S9) show that in this approximation, as for the DFT result, show that **II** becomes less stable relative to the other phases as the temperature increases. The similar trend in free energy for **III** and **IV** may reflect the similar packing motifs found in these structures.

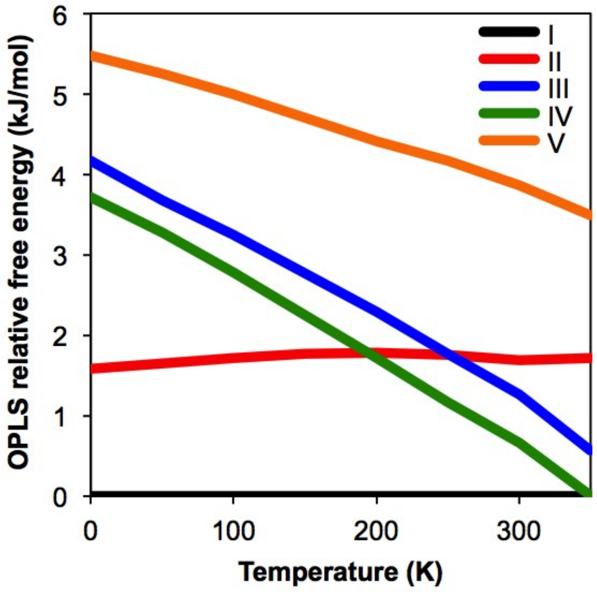

Fig. S9 Relative free energy of coumarin polymorphs using the classical harmonic approximation at temperatures from 50 – 350 K. Cell vectors are from MD simulations at each temperature.

Following our previous work,[18,19,20] we used thermodynamic integration (TI) to compute the anharmonic relative free energy difference between coumarin polymorphs. This approach was divided in two steps: i) identification of a continuous path between two structures and ii) calculating the free energy difference along the path.

Steered molecular dynamics simulations were used to interconvert the coumarin polymorphs.[21] First we identified a molecular match between polymorph packings, as shown in Fig. S10. To pull the entire supercell system, 8 molecules were required as a reference, expanding the crystalline unit cells. We pulled each molecule with a set collective variables (CVs) based on center of mass distances and relative quaternions[22] between molecules. The CVs are based on the reference molecules and replicated in the rest of the supercell to conserve the crystal order while allowing local fluctuations.

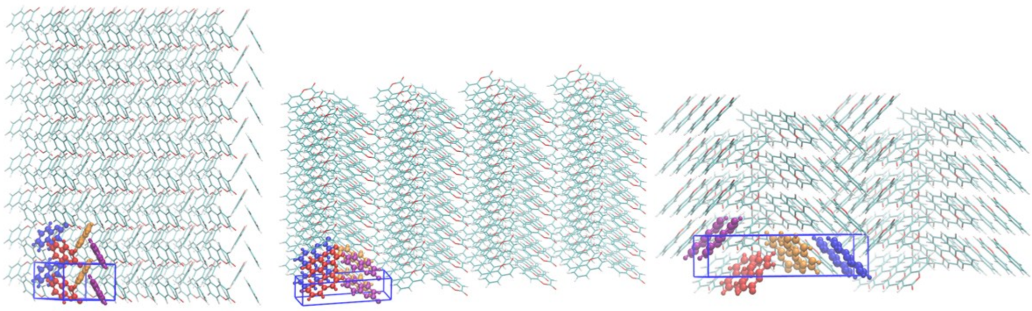

Fig. S10 Simulated supercell in steered MD. Reference molecules used to compute the set of CVs composed by center of mass distance and relative quaternions between molecules are shown in red, blue, purple, and gold. The blue box indicates the unit cell. The steered MD path interconverts each set of colored molecules for coumarin **I** (left), coumarin **II** (middle), and coumarin **V** (right).

Once a transition path was obtained, the relative free energy between the two polymorphs was determined by calculating the free energy gradients, i.e. mean forces, for the set of CVs and h-matrix visited along the steered MD simulation.[13,20] Integrating the mean force along the path yields the relative free energy difference between forms **I**, **II** and **V**.

We used the TI technique at 100, 200, and 300 K obtaining a smaller relative free energy difference at lower temperatures (Table S4). Unlike the harmonic approximation results, the relative free energy difference for both **II** and **V** increases with temperature, indicating that anharmonic affects are likely to be important in obtaining a stability ranking that is consistent with experimental results. As a check for the error of our method at room temperature, we performed an independent computation for the relative free energy between **II** and **V** for 300 K, obtaining a result consistent with respect to the free energy difference with **I** at the same temperature. Therefore, although the error in classical energies and forces due to the use of static charges results in relative energies that are too large, the predicted relative stability of coumarin **V** is correct when anharmonic effects are included.

Table S4 Relative free energy differences for select coumarin polymorphs based on steered MD (kJ/mol). “n/d” indicates values that were not determined.

Temperature (K)	**I → II**	**I → V**	**II → V**
100	3.2 ± 0.7	15.0 ± 1.6	n/d
200	3.9 ± 0.7	15.5 ± 1.6	n/d
300	4.5 ± 0.7	16.0 ± 1.6	11.6 ± 0.7

REFERENCES

1 A. O. Lyakhov, A. R. Oganov, H. T. Stokes and Q. Zhu, *Comput. Phys. Comm.*, 2013, **184**, 1172.

2 Q. Zhu, A. R. Oganov, C. W. Glass and H. T. Stokes, *Acta. Cryst.*, 2012, **B68**, 215.

3 S. L. Price, M. Leslie, G. W. A. Welch, M. Habgood, L. S. Price, P. G. Karamertzanis and G. M. Day, *PhysChemPhys*, 2010, **12**, 8478.

4 D. S. Coombes, S. L. Price, D. J. Willock, M. Leslie, *J. Phys. Chem.*, 1996, **100**, 7352-7360

5 A. J. Stone, M. Alderton, *Molecular Physics*, 1985, **56**, 1047-1064

6 M. J. Frisch, G. W. Trucks, H. B. Schlegel, G. E. Scuseria, M. A. Robb, J. R. Cheeseman, G. Scalmani, V. Barone, B. Mennucci, G. A. Petersson, H. Nakatsuji, M. Caricato, X. Li, H. P. Hratchian, A. F. Izmaylov, J. Bloino, G. Zheng, J. L. Sonnenberg, M. Hada, M. Ehara, K. Toyota, R. Fukuda, J. Hasegawa, M. Ishida, T. Nakajima, Y. Honda, O. Kitao, H. Nakai, T. Vreven, J. A. Jr. Montgomery, J. E. Peralta, F. Ogliaro, M. Bearpark, J. J. Heyd, E. Brothers, K. N. Kudin, V. N. Staroverov, R. Kobayashi, J. Normand, K. Raghavachari, A. Rendell, J. C. Burant, S.

S. Iyengar, J. Tomasi, M. Cossi, N. Rega, J. M. Millam, M. Klene, J. E. Knox, J. B. Cross, V. Bakken, C. Adamo, J. Jaramillo, R. Gomperts, R. E. Stratmann, O. Yazyev, A. J. Austin, R. Cammi, C. Pomelli, J. W. Ochterski, R. L. Martin, K. Morokuma, V. G. Zakrzewski, G. A. Voth, P. Salvador, J. J. Dannenberg, S. Dapprich, A. D. Daniels, O. Farkas, J. B. Foresman, J. V. Ortiz, J. Cioslowski and D. J. Fox, Gaussian 09, version D.01, Gaussian, Inc., Wallingford, CT, 2009.

7 G. Kresse and J. Furthmüller, *Phys. Rev. B*, 1996, **54**, 11169.

8 J. P. Perdew, K. Burke and M. Ernzerhof, *Phys. Rev. Lett.*, 1996, **77**, 3865.

9 C. Adamo and V. Barone, *J. Chem. Phys.*, 1999, **110**, 6158.

10 B. P. van Eijck and J. Kroon, *J. Comput. Chem.*, 1999, **20**, 799.

11 W. L. Jorgensen, D. S. Maxwell and J. Tirado-Rives *J. Am. Chem. Soc.*, 1996, **118**, 11225.

12 J. A. Chisholm and S. J. Motherwell, *Appl. Crystallogr.,* 2005, **38**, 228.

13 M. E. Tuckerman, D. Yarne, S. O. Samuelson, A. L. Hughes and G. J. Martyna, *Comput. Phys. Commun.*, 2000, **128**, 333.

14 G. J. Martyna, M. L. Klein and M. Tuckerman, *J. Chem. Phys.*, 1992, **97**, 2635.

15 G. J. Martyna, D. J. Tobias and M. L. Klein, *J. Chem. Phys.*, 1994, **101**, 4177.

16 G. J. Martyna, M. E. Tuckerman, D. J. Tobias and M. L. Klein, *Mol. Phys.*, 1996, **87**, 1117.

17 M. E. Tuckerman, J. Alejandre, R. López-Rendón, A. L. Jochim and G. J. Martyna, *J. Phys. A Math. Gen.*, 2006, **39**, 5629.

18 T-Q. Yu, P-Y., Chen, M. Chen, A. Samanta, E. Vanden-Eijnden and M. E. Tuckerman, *J. Chem. Phys.*, 2014, **140**, 214109.

19 Q. Zhu, A. Shtukenberg, D. Carter, T-Q. Yu, J-X. Yang, M. Chen, P. Raiteri, A. Oganov, B. Pokroy, I. Polishschuk, P. Bygrave, G. Day, A. Rohl, M. Tuckerman and B. J. Kahr, *Am. Chem. Soc.*, 2016, **138**, 4881.

20 E. Schneider, L. Vogt and M. E. Tuckerman, *Acta Cryst.*, 2016, **B72**, 542.

21 R. Elber, *J. Chem. Phys.* 1990, **93**, 4312.

22 E. A. Coutsias, C. Seok, K. A. Dill, *J. Comp. Chem.*, 2004, **25**, 1849.
